# Electrothermally‐Driven Ultrafast Chemical Modulation of Multifunctional Nanocarbon Aerogels

**DOI:** 10.1002/smll.202404364

**Published:** 2024-08-08

**Authors:** Dong Xia, Qun Li, Jamie Mannering, Yi Qin, Heng Li, Yifei Xu, Ashiq Ahamed, Wenyu Zhou, Alexander Kulak, Peng Huang

**Affiliations:** ^1^ Department of Engineering Science University of Oxford Oxford OX1 3PJ UK; ^2^ School of Chemistry University of Leeds Leeds LS2 9JT UK; ^3^ School of Chemistry and Chemical Engineering Chongqing University Chongqing 400044 China; ^4^ Key Laboratory of Estuarine Ecological Security and Environmental Health, Tan Kah Kee College Xiamen University Zhangzhou 363105 China; ^5^ State Key Laboratory of Molecular Engineering of Polymers, Department of Macromolecular Science Fudan University Shanghai 200438 China; ^6^ Henry Royce Institute The University of Manchester Manchester M13 9PL UK

**Keywords:** catalytic desulfurization, DFT calculations, flash Joule‐heating, membrane filtration, nanocarbon aerogels, ultrahigh‐temperature

## Abstract

Ultrahigh‐temperature Joule‐heating of carbon nanostructures opens up unique opportunities for property enhancements and expanded applications. This study employs rapid electrical Joule‐heating at ultrahigh temperatures (up to 3000 K within 60 s) to induce a transformation in nanocarbon aerogels, resulting in highly graphitic structures. These aerogels function as versatile platforms for synthesizing customizable metal oxide nanoparticles while significantly reducing carbon emissions compared to conventional furnace heating methods. The thermal conductivity of the aerogel, characterized by Umklapp scattering, can be precisely adjusted by tuning the heating temperature. Utilizing the aerogel's superhydrophobic properties enables its practical application in filtration systems for efficiently separating toxic halogenated solvents from water. The hierarchically porous aerogel, featuring a high surface area of 607 m^2^ g^−1^, ensures the uniform distribution and spacing of embedded metal oxide nanoparticles, offering considerable advantages for catalytic applications. These findings demonstrate exceptional catalytic performance in oxidative desulfurization, achieving a 98.9% conversion of dibenzothiophene in the model fuel. These results are corroborated by theoretical calculations, surpassing many high‐performance catalysts. This work highlights the pragmatic and highly efficient use of nanocarbon structures in nanoparticle synthesis under ultrahigh temperatures, with short heating durations. Its broad implications extend to the fields of electrochemistry, energy storage, and high‐temperature sensing.

## Graphical abstract

The excellent electrical conductivity of nanocarbon aerogels enables them to be Joule‐heated to ultrahigh temperatures of ≈3000 K, which results in improved physicochemical properties, including superhydrophobicity and the ability to serve as flash microfurnaces for nanocatalyst synthesis. These enhancements are demonstrated through proof‐of‐concept experiments involving efficient oil–water separation and electrochemically‐driven flash synthesis of MoO_2_ nanoparticles for exceptional catalytic desulfurization.

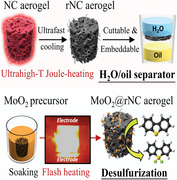

## 1 Introduction

Graphene and carbon nanotubes are prominent allotropes of carbon, renowned for their exceptional properties including high surface area, outstanding electrical and thermal conductivities, and remarkable mechanical strength.^[^ ^1^, ^2^, ^3^, ^4^, ^5^ ^]^ However, harnessing these characteristics on a bulk scale through wet‐chemical processes presents challenges due to the inherently hydrophobic nature of the graphitic lattice. One effective strategy for modifying graphitic nanocarbons involves acid oxidation to fabricate their oxidized formats such as graphene oxide (GO) and oxidized carbon nanotubes (oCNTs), which introduces polar surface functionalities. These polar functionalities enhance dispersion within polar solvents, making oxidized nanocarbons highly water‐processable.^[^ ^6^ ^]^ By dispersing and assembling GO and oCNTs, followed by solvent elimination, it becomes possible to engineer low‐density 3D porous aerogels. These aerogels offer a pathway to realize nanoscale graphitic properties within the bulk phase, providing attributes that are inaccessible to their powder analogs.^[^ ^7^ ^]^

The current challenges in nanocarbon (NC) aerogel fabrication primarily concern the development of efficient methods for chemically modifying the aerogel framework, especially during the reduction stage aimed at restoring graphitic properties. Conventional thermal reduction approaches tend to be slow and can result in undefined end products, often leading to restacking within NC structures, particularly evident when reducing GO, thus compromising their nanoscale properties.^[^ ^8^ ^]^ To tackle this challenge, several strategies have emerged, such as incorporating spacers like melamine resin or hybridizing different types of NCs (e.g., GO and oCNTs), which have shown promise in addressing these limitations.^[^ ^9^, ^10^ ^]^

The adoption of local resistive electrical heating, also known as Joule‐heating, represents a promising alternative approach either independently or in conjunction with spacers. This method enables rapid thermal reduction with high efficiency,^[^ ^11^, ^12^, ^13^ ^]^ effectively preventing restacking while preserving surface area within the aerogel structure. The resulting aerogels offer unique advantages, including increased surface area, tailored hierarchical porosity that enhances mass transport through the pores, and efficient recovery from reactions without the necessity for additional separation processes.^[^ ^14^, ^15^ ^]^ Recent advancements in ultrahigh‐temperature Joule‐heating, capable of reaching temperatures up to 3000 K for NC films and fibers, have significantly broadened the scope of these materials. However, reports detailing this application to NC aerogels remain limited in the literature. It is worth noting that ultrahigh‐temperature Joule‐heating can be employed for the rapid synthesis of inorganic nanoparticles (NPs), enabling additional functionalization of NC aerogels and expanding their versatility.^[^ ^16^, ^17^ ^]^

Our previous research demonstrated the feasibility of fabricating GO‐assembled aerogels using ultrahigh‐temperature Joule‐heating.^[^ ^18^, ^19^ ^]^ To substantiate the universality of the electrothermally‐driven ultrahigh‐temperature Joule‐heating methodology, we further fabricated NC aerogels assembled by GO and oCNT, which exhibited internal microstructure diversities and reduced restacking tendencies due to the oCNT functioning as the spacer. Additionally, this work explored the practical applications of hybridized NC aerogels prepared using ultrahigh‐temperature Joule‐heating. Our investigation delved into the impact of restacking, graphitization, structural stability, and the carbon footprint of the fabrication process. Furthermore, we leveraged the aerogel framework as a versatile platform for synthesizing inorganic nanoparticles with adjustable compositions and catalytic functionalities. We conducted comprehensive characterizations of these hybrid aerogels to gain insights into their structure and physiochemical properties. These characterizations were then correlated with their catalytic desulfurization efficiency, and the underlying mechanisms were elucidated through theoretical calculations.

## 2 Results and Discussion

### 2.1 Fabrication of Hybrid Aerogels

Our investigation embarked on the fabrication of a 3D macroscopic NC aerogel with electrical conductivity to enable self‐resistive heating. We employed a hydrothermal synthesis method to produce these NC aerogels, as illustrated in Figure S1 (Supporting Information).^[^ ^20^, ^21^ ^]^ To optimize the aerogel's performance, we incorporated oCNTs (avg. length 2 µm and width 18 nm) as steric bridges, akin to graphene intercalation, which effectively prevented GO restacking during partial chemical reduction. GO served as the primary network former, exhibiting lateral dimensions of 0.8 × 0.8 µm. During the hydrothermal process, well‐dispersed nanocarbons underwent partial reduction facilitated by L‐ascorbic acid, leading to spontaneous self‐assembly into a porous nanocarbon network through non‐covalent van der Waals interactions. This process resulted in the formation of a stable hybrid NC hydrogel network, which, upon freeze–drying, produced free‐standing and structurally intact NC aerogels (Figure S2, Supporting Information). These aerogels served as precursors for subsequent Joule‐heating studies.

**Figure 1:**
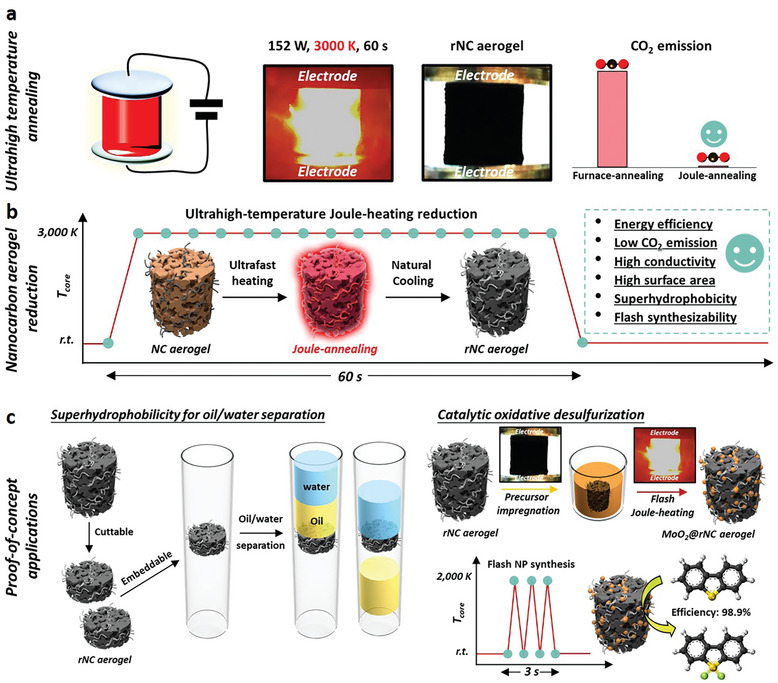
a) Schematic illustrating the creation of physicochemically enhanced rNC aerogel through ultrahigh‐temperature Joule‐heating, showcasing minimal CO_2_ emissions. b) Diagram of the ultrahigh‐temperature Joule‐heating reduction process applied to rNC aerogel, resulting in enhanced various physiochemical properties. c) Schematic of the applications of the rNC aerogel, including oil/water separation and flash synthesis of MoO_2_@rNC aerogel for effective catalytic oxidative desulfurization.

The efficacy of oCNTs in mitigating GO restacking is validated through a comparison of the powder X‐ray diffraction (XRD) patterns (002 diffraction peak analysis) of the dried hybrid NC aerogel with a GO aerogel fabricated under identical conditions but without oCNTs (Figure S3 and Table S1, Supporting Information). The broad (002) XRD peak observed for the hybrid oNC aerogel and the reduced number of sheets per crystallographic domain (five layers for the NC aerogel and eight layers for the GO aerogel) demonstrated decreased GO stacking during hybrid aerogel assembly. To enhance the physicochemical properties of the NC aerogels, we explored thermal treatment at elevated temperatures, aiming to promote the formation of more graphitic nanocarbon. Conventional thermal treatment methods typically involve energy‐intensive thermal annealing in furnaces. In contrast, our study introduces an innovative approach utilizing ultrahigh‐temperature Joule‐heating for a brief period, referred to as flash Joule‐heating (distinguished from conventional Joule‐heating by its rapid heating rate and short duration, **Figure** **1**a), to restore the physicochemical properties of oxidized NC structures (Figure 1b).

**Figure 2:**
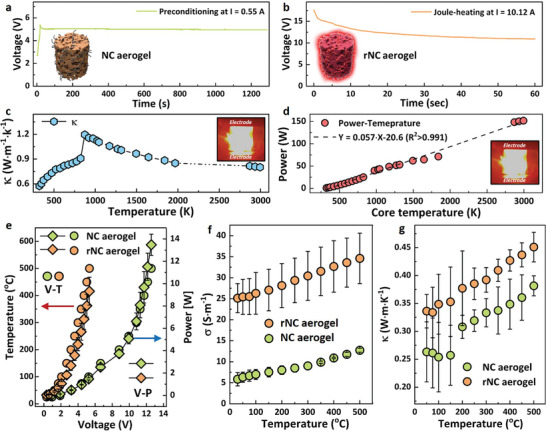
a) Preconditioning of a NC aerogel precursor via Joule‐heating at *I* = 0.55 A to remove water and obtain the NC aerogel. b) Ultrahigh‐temperature Joule‐heating of NC aerogel at *I* = 10.12 A for 60 s to synthesize rNC aerogel. Insert: Digital photo of NC aerogel during Joule‐heating. c) Umklapp scattering analysis illustrating the correlation between thermal conductivity and Joule‐heating temperature of rNC aerogel. d) Power input as a function of Joule‐heating temperature for rNC aerogel. e) Relationship between voltage, input power, and Joule‐heating temperature for NC aerogel and rNC aerogel. Electrical conductivity f) and thermal conductivity g) as functions of Joule‐heating temperature for NC aerogel and rNC aerogel.

Monitoring the Joule‐heating temperature of an NC aerogel at a low current (0.55 A) was achieved using thermocouples. Maintaining this Joule‐heating condition for 20 min is crucial for removing residual water and stabilizing the NC aerogel's Joule‐heating capabilities, as evidenced by the consistent voltage observed during extended Joule‐heating sessions (**Figure** **2**a). Flash Joule‐heating at a higher current (10.12 A for 60 s, Figure 2b) generated ultrahigh temperatures in the aerogel, resulting in intensive black‐body radiation phenomenon (Figure 2c, inset) and yielding a reduced NC (rNC) aerogel with significantly improved physiochemical properties. Notably, the energy consumption for annealing the aerogels by Joule‐heating was ultralow, with heating periods of 10, 30, and 60 s requiring only 4.12 × 10^−4^, 1.25 × 10^−3^, and 2.50 × 10^−3^ kWh, respectively, highlighting the high energy efficiency of ultrahigh‐temperature Joule‐heating for annealing NC aerogel.

Challenges arise when subjecting the aerogel to such high temperatures (up to 3000 K), as the temperature in the aerogel center could not be directly recorded. To address this, we have previously developed a methodology combining a 1D heat conduction model with power law analysis for accurate core temperature estimation based on Umklapp scattering analysis (detailed methods and analyses see Figures S4–S8, Supporting Information).^[^ ^18^, ^19^ ^]^ Umklapp scattering analysis (Figure 2c)^[^ ^22^ ^]^ elucidates that the rNC aerogel could reach temperatures of up to ≈3000 K at 10.12 A (Figures S6–S8, Supporting Information), far exceeding conventional furnace temperatures (typically below 1573 K).^[^ ^23^ ^]^ The high linearity observed in the power‐temperature relationship (*R* ^2^ > 0.991, Figure 2d) validated the methodology's robustness in estimating aerogel core temperatures and demonstrated the capability of Joule‐heating to achieve precise temperature control.

Environmental benefits of our flash Joule‐heating approach were highlighted through life cycle analysis, showing significant reductions in global warming potential (GWP) compared to conventional furnace heating methods (Figure S9, Tables S2 and S3, Supporting Information). Flash Joule‐heating exhibited a GWP that was 959 and 1148 times lower than conventional furnace heating methods 1 (3000 K, 60 s) and 2 (1273 K, 2 h), respectively, primarily due to reduced electricity consumption. Conventional furnace heating incurs considerable energy consumption to raise the temperature of the aerogel and its surroundings, while Joule‐heating, in contrast, directly heats the aerogel to the desired temperature with minimal energy loss. The GWP analysis highlighted the advantages of employing flash Joule‐heating for annealing nanocarbon‐based bulk materials, resulting in enhanced properties and paving the way for diverse applications (Figure 1c), as elaborated in subsequent sections.

### 2.2 Physicochemical Properties of Hybrid Aerogels

After successfully synthesizing the aerogels, we further investigated their physicochemical properties. The rNC aerogel (density of 15.14 mg cm^−3^, Figure S16, Supporting Information) demonstrates remarkable energy efficiency, requiring significantly lower voltage (Figure S10, Supporting Information) and power to achieve equivalent Joule‐heating temperatures compared to the NC aerogel (Figure 2e). Additionally, it exhibits impressive electrical conductivity (25 S m^−1^, Figure 2f) and thermal conductivity (0.33 W m K^−1^, Figure 2g), representing substantial improvements over the original NC aerogel. These enhancements can be attributed to the restored graphitic nanostructures, which facilitate the mobilization of both electrons and phonons. The superiority of the rNC aerogel's electrical conductivity can be elucidated through the fitting of the 3D variable hopping model, revealing its proficiency in enabling uniform resistive heating.^[^ ^15^ ^]^ This uniformity is further supported by a high coefficient value (*R* ^2 ^> 0.996, Figure S11, Supporting Information) and the even color distribution in the infrared thermal image (Figure S12, Supporting Information). The transformation of the rNC aerogel into a semiconducting material is notable, with bandgap values shifting from 85.0 to 22.2 meV, as determined by the Arrhenius thermal activation model (Figure S13, Supporting Information).^[^ ^22^ ^]^ This aligns with the observed electrical conductance parameters (Figure 2f).

The flash Joule‐heating process yields additional physicochemical properties, including improved surface area confirmed by N_2_ sorption measurements (Figure S14, Supporting Information), enhanced structural stability affirmed by thermogravimetric analysis (Figure S15, Supporting Information), and reduced aerogel density as a function of heating duration (Figure S16, Supporting Information). Additionally, we observed a considerable upgrade in NC quality, as revealed by X‐ray photoelectron spectroscopy where the at.% of C increased from 94.6% in NC aerogel to 99.3% in rNC aerogel (Figures S17–S20, Supporting Information).

**Figure 3:**
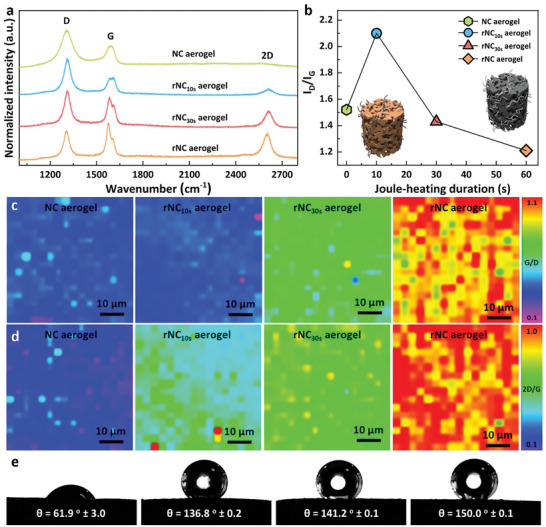
Raman spectra and contact angle profiles of NC aerogel, rNC aerogel_10s_ aerogel, rNC aerogel_30s_ aerogel and rNC aerogel. a) Raman spectra collected at the wavelength of 785 nm, in which the D band is known as the defect band that represents a ring breathing mode from sp^2^ carbon rings, G band is a primary in‐plane vibrational mode that represents the planar configuration sp^2^ bonded carbon that constitutes graphene and the 2D band is formed by a second‐order overtone of a different in‐plane vibration. b) I_D_/I_G_ value as a function of Joule‐heating duration. Raman mapping of c) I_G_/I_D_ values and d) I_2D_/*I*_G_ values collected at the wavelength of 785 nm. e) Contact angle profiles.

These substantially enhanced characteristics prompted us to investigate whether further improvements could be achieved by fine‐tuning the flash Joule‐heating duration. This parameter plays a pivotal role in governing the extent of graphitic restoration, as confirmed by the drastically varied Raman spectra (**Figure** **3**a). Specifically, three pronounced bands emerged in the collected Raman spectra, where the peak positions located at ≈1313, ≈1578, and ≈2602 cm^−1^ correspond to the D band (known as the defect band representing a ring‐breathing mode from sp^2^ carbon rings), the G band (a primary in‐plane vibrational mode representing the planar configuration of sp^2^ bonded carbon constituting graphene), and the 2D band (formed by a second‐order overtone of a different in‐plane vibration), respectively.^[^ ^24^ ^]^ The I_D_/I_G_ ratio is widely adopted to assess the graphiticity of defective nanocarbon material.^[^ ^19^ ^]^ Among various heating durations, the rNC aerogel obtained with 60 s Joule‐heating underwent substantial crystallinity restoration. For simplicity, we denote the aerogels produced with 10, 30, and 60 s Joule‐heating as rNC_10s_ aerogel, rNC_30s_ aerogel, and rNC aerogel, respectively. The I_D_/I_G_ value increased in the rNC_10s_ aerogel and subsequently dropped substantially in both the rNC_30s_ aerogel and rNC aerogel (Figure 3b).^[^ ^7^ ^]^

This nonlinear I_D_/I_G_ ratio trend fluctuation follows well with the Tuinstra–Koenig relationship,^[^ ^25^ ^]^ which points out that highly defective graphitic materials (such as GO) will emerge an opposing evolution of I_D_/I_G_ ratio with an increasing graphitization. This highlights the effectiveness of rapid electrothermal reduction, expected to enhance NC junction interactions and improve the electrical percolation of the rNC aerogel (see Figure 2f for conductivity data). Raman mapping was employed to visualize the 2D uniformity of graphitic recrystallization in the resultant aerogels and assess the homogeneity of thermal reduction under Joule‐heating. This technique depicted varying degrees of graphiticity as different colors, with blue signifying regions with high defectiveness (small I_G_/I_D_ or I_2D_/I_G_ values), while red indicated areas with high graphitic nature (high I_G_/I_D_ or I_2D_/I_G_ values, Figures 3c,d).^[^ ^26^, ^27^ ^]^ The maps offered high‐resolution depictions of the graphiticity across the aerogel surface. The 2D map collected from the core of the aerogel revealed excellent graphitic uniformity and complete thermal reduction within 60 s at 3000 K, attesting to the efficiency of flash Joule‐heating in the fabrication of rNC aerogels.

A key outcome of the flash Joule‐heating process was the achievement of superhydrophobicity in the rNC aerogel, as indicated by a contact angle of 150°.^[^ ^28^ ^]^ This stands in stark contrast to the NC aerogel, which features a hydrophilic nature with a contact angle of only 61.9° (Figure 3e). These contact angle measurements align with the Raman spectra data (Figure 3a,b), affirming that the enhanced hydrophobicity of the resulting rNC aerogel stems from improved graphitic features (Figure 3e). Validation of the enhanced porosity is evident in scanning electron microscopy (SEM) images of the rNC aerogel, before and after thermal reduction (Figures S21b, Supporting Information, avg. macropore size 1–2 µm). The open porosity remains intact during electrical reduction, affirming the efficiency of the rapid reduction treatment (Figure S21a for NC aerogel and Figure S21b for rNC aerogel, Supporting Information). Higher magnification images further corroborate the bridging functionality of the CNT building blocks (Figures S22 and S23, Supporting Information). The high specific surface area and open hierarchical porosity of the rNC aerogel are desirable attributes for effective functionalization with inorganic metallic NPs (Table S4, Supporting Information). This leads to improved catalytic desulfurization performance (vide infra). The wider implications of unaltered porosity profiles during reduction imply that porosity can be tailored and maintained throughout the aerogel fabrication process. Transmission electron microscopy (TEM) images of the rNC aerogel reveal a homogenous mixing of the constituent NC building blocks (Figure S21c before reduction and Figure S21d after reduction, Supporting Information). High magnification TEM images (Figures S21e,f and S24, Supporting Information) further underscore the absence of amorphous carbon coating the NCs, confirming the effective reduction achieved through rapid electrical heating at ultrahigh temperatures.

### 2.3 Applications of Hybrid Aerogels

After characterizing the hybrid aerogels, we turned our attention to exploring their practical applications. Rapid electrical reduction enabled adjustment of the NC aerogel's hydrophobicity, which depended on the electrical power input and the corresponding reduction temperature. Notably, the rNC aerogel, reduced at 3000 K for 60 s, demonstrates superhydrophobicity (*θ* = 150 °C, Figure 3e). This property in conjunction with an impressive absorption capacity for organic solvents (as seen in colored solvents absorbed by the bulk rNC aerogel in Figures S25 and S26, Supporting Information), was leveraged in an aerogel membrane application.

**Figure 4:**
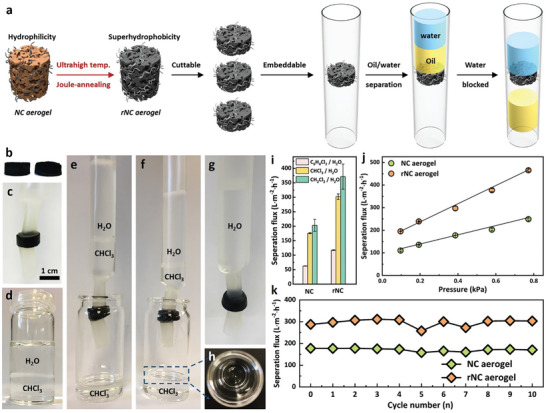
a) Schematic demonstration of the fabrication process for the oil/water separator using NC and rNC aerogel. Digital photos of NC aerogel and rNC aerogel b), and rNC aerogel enclosed in heat shrinkage tubing as an oil/water separator c). d) Photograph of H_2_O and CHCl_3_ mixtures in a glass vial with a volume ratio of 1:1 (10 mL). Custom‐made oil/water separators using NC aerogel e) and rNC aerogel f) for CHCl_3_/H_2_O separation. g) Visual demonstration of water phase blockage by superhydrophobic rNC aerogel upon completion of separation. h) Photograph of the separated CHCl_3_ phase in a glass vial. i) Comparison of NC aerogel and rNC aerogel performance in separating different oil/water mixtures. j) Pressure‐induced separation influx of CHCl_3_/H_2_O mixtures. The pressure was adjusted based on the volumes of CHCl_3_ and H_2_O while maintaining total volume unchanged. k) Recycling performance comparison of NC aerogel and rNC aerogel for CHCl_3_/H_2_O mixture separation.

In this application (**Figure** **4**a), the rNC aerogel was embedded within a column (Figure 4b) and filled with a halogenated solvent‐water mixture (vol. ratio 1:1, 10 mL, Figure 4c). When tested with a model mixture of chloroform‐water (Figure 4d), rapid and effective separation occurred (as depicted in time‐lapse Figure 4e–g), showcasing its potential for large‐scale volume filtration under gravitational force (Figure 4h).^[^ ^29^ ^]^ Further, when subjected to elevated pressures, separation flux increased (475 L m^−2^ h^−1^ at 0.8 kPa, Figure 4j, vs the initial 200 L m^−2^ h^−1^), affirming the mechanical strength of the aerogel. Similar separation performance was observed with other mixtures (dichloromethane and 1,4‐dichlorobenzene, Figure 4i), highlighting the rNC aerogel's functional capability, reliable separation repeatability (Figure 4k), and robust mechanical strength (observed through resistance to deformation during filtration and the ability to cut the aerogels into pre‐defined shapes, Figure 4b). Additionally, its superhydrophobic nature opens avenues for exploring applications related to the lotus effect without the need for surface asperities, especially in structural engineering.^[^ ^30^ ^]^

**Figure 5:**
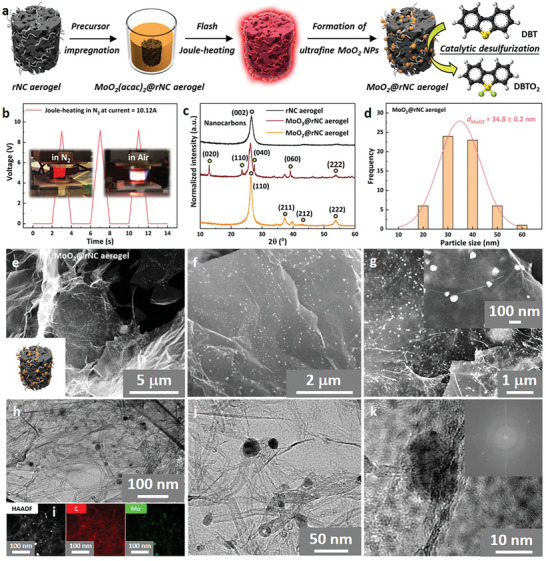
a) Schematic illustration of the flash synthesis process for the MoO_2_@rNC aerogel catalyst used in catalytic oxidative desulfurization, involving soaking in MoO_2_(acac)_2_‐contained chloroform solution, ambient drying, and flash Joule‐heating to produce the embedded MoO_2_ nanoparticles. b) Joule‐heating voltage as a function of heating time for producing MoO_2_@rNC aerogels at a current of *I* = 10.12 A (1 s, pulsed 3 times). Inset: Digital photos of MoO_2_(acac)_2_‐impregnated rNC aerogels during Joule‐heating in N_2_ and air atmospheres. c) XRD patterns of rNC aerogel, MoO_3_@rNC aerogel fabricated in air atmosphere, and MoO_2_@rNC aerogel fabricated in N_2_ atmosphere. e) Particle size analysis based on SEM image f. e–g) SEM images of MoO_2_@rNC aerogels at different magnifications. h) TEM image of MoO_2_@rNC aerogel. i) HR‐TEM image of MoO_2_@rNC aerogel with corresponding elemental maps. j,k) Magnified HR‐TEM images of MoO_2_@rNC aerogel, including the Fast Fourier transform pattern of MoO_2_ nanoparticles embedded in image k.

Another significant advantage of the electrically conductive aerogels was their rapid heating and cooling capabilities, which were harnessed in the following section for the fabrication of hybrid NC aerogels with embedded functional inorganic NPs. The rNC aerogel was modified with MoO_2_ NPs (denoted as MoO_2_@rNC aerogel, **Figure** **5**a)^[^ ^31^ ^]^ through a straightforward solution phase impregnation method using MoO_2_(acac)_2_ as the precursor. Shock‐decomposition via electrical heating (3 s at ≈2000 K, Figure 5b) was used to selectively form molybdenum dioxide NPs (Mo(IV)O_2_) in nitrogen or molybdenum trioxide NPs (Mo(VI)O_3_) in air with no visible aerogel shrinkage (see XRD patterns in Figure 5c). Both these NP types are highly active chemical and electrochemical catalysts, with MoO_2_ demonstrating the most catalytic activity and thus serving as a model catalyst for further characterization.^[^ ^32^ ^]^

The shock‐decomposition process facilitated the even distribution of well‐sized MoO_2_ NPs across the entire NC surface (see SEM images in Figure 5e–g), with average particle sizes of 34.8 nm (determined through SEM size distribution analysis, Figure 5f). These MoO_2_ NPs exhibited crystallinity within a monoclinic crystal phase, as confirmed by TEM images (Figures 5h–k) and electron scattering determined within the Fourier‐transformed spectrum (Figure 5k inset), as well as XRD patterns (Figure 5c). High‐angle annular dark‐field (HAADF) imaging (Figure 5i), energy‐dispersive X‐ray (EDX) spectroscopic mapping (Figures 5i) and XPS spectra (Figure S27, Supporting Information) were employed for elemental composition verification.

**Figure 6:**
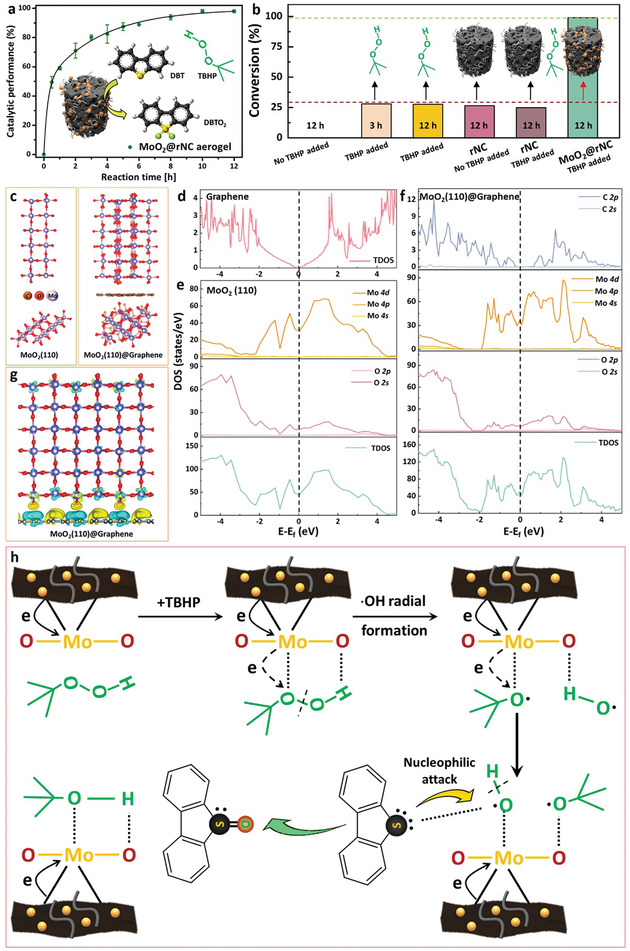
a) Catalytic oxidative performance of MoO_2_@rNC aerogel catalysts in desulfurizing DBT, with accompanying schematics illustrating catalytic reactions on the surface of MoO_2_ nanoparticles. b) Comparative catalytic oxidative performance of DBT under various catalysts and experimental conditions. TBHP: tert‐butyl hydroperoxide. c) Crystal structures of MoO_2_(110) surfaces and MoO_2_(110)@graphene surfaces along different directions. d) Total density of states (TDOS) of graphene. e) Partial density of states (PDOS) of MoO_2_(110) surface. f) PDOS profiles of MoO_2_(110)@graphene. The Fermi level is indicated by the dotted line. g) Charge density differences of MoO_2_(110)@graphene, where yellow (blue) areas represent regions of charge density accumulation (depletion) at an isosurface corresponding to 0.002 |e|/Å^3^. h) Schematic illustration of the catalytic mechanism of MoO_2_@rNC aerogel catalysts for the oxidative desulfurization of DBT. DBT: dibenzothiophene.

The catalytic oxidative desulfurization of dibenzothiophene (DBT, a common organosulfur compound found in fuels) served as a test case for the MoO_2_@rNC aerogel. Oxidative transformation of organosulfur compounds into sulfones enables better separation due to increased polarity and is essential for mitigating environmental concerns (*i.e*., formation of acid rain) related to sulfur‐containing compounds in transportation fuels. The DBT was efficiently converted into dibenzothiophene 5,5‐dioxide (DBTO_2_), catalyzed by supported MoO_2_ NPs (**Figure** **6**a inset), and exhibited remarkable catalytic performance with a 98.9% conversion of DBT into DBTO_2_ within 10 h (Figure 6a). Such remarkable performances were contributed from the uniformly and densely distributed flash‐synthesized MoO_2_ NPs on the surface of nanocarbons (as observed in both SEM and TEM images in Figure 5), leading to the exposure of more active sites for DBT conversion. In addition, the distinguished catalytic oxidative DBT conversion capability from the flash‐synthesized MoO_2_ nanocatalysts was corroborated by various experimental conditions (Figure 6b). Standardized metrics indicated an outstanding catalyst efficiency in terms of both turnover and rate (capacity: 69 g S g^−1^ MoO_2_; adjusted capacity: 0.34 µmole_DBTO2_⋅S^−1^ m^−2^ MoO_2_). This method represents a sustainable alternative to conventional hydrodesulfurization and demonstrates the viability of oxidative desulfurization with low‐cost, scalable catalytic materials. Notably, the MoO_2_@rNC aerogel developed within this work exhibits comparable catalytic DBT conversion capacity to recent high‐performance heterojunction MoO_2_/*g*‐boron–nitride catalyst,^[^ ^33^ ^]^ MoO_x_ catalyst supported on boron nitride,^[^ ^34^ ^]^ and MoO_2_ in carbon nanoreactors,^[^ ^32^ ^]^ potentially due to a fundamental electronic interaction between the MoO_2_ catalyst and the graphitic support, resulting in pronounced catalytic activity.

### 2.4 DFT Calculations

To validate the aforementioned assumptions and elucidate the catalytic mechanism of the supported MoO_2_ catalyst, we performed computational modeling using density functional theory (DFT).^[^ ^35^ ^]^ With insights drawn from both XRD (reflecting bulk crystallinity) and TEM (representing surface crystallinity), we successfully simulated and validated the existence of the MoO_2_(110) phase (Figure 6c). As a model substrate support, graphene was chosen, acting as a representative system for both reduced graphene oxide and multi‐walled carbon nanotubes (Figure S28, Supporting Information).

The density of states (DOS) for graphene, MoO_2_(110) and graphene‐supported MoO_2_(110) (MoO_2_(110)@graphene) was assessed through DFT calculations at ambient temperature. The total density of states (TDOS) of graphene reveals the well‐known behavior of a zero‐bandgap semimetal (Figure 6d). Meanwhile, the conduction band of MoO_2_ emerged from the contributions of Mo 4*d* and O 2*p* orbitals (Figure 6e), giving rise to overlapping valence and conduction bands (i.e., metallic). The metallicity of MoO_2_(110)@graphene (Figure 6f) exhibited a substantial increase, indicated by the downshift of the partial density of states (PDOS) of Mo and O atoms along the Fermi level. This shift indicated a profound electron transfer from graphene to Mo and O atoms. The electron transfer is further confirmed by the charge density difference profile (Figure 6g), substantiating the considerable electron transfer from graphene to the metal oxides, thus leading to the presence of active Mo species with a higher electron density and in turn highly active.

Consequently, the adjacent O atoms in tert‐butyl hydroperoxide (TBHP) acquired a higher electron density (Figure 6h), promoting the cleavage of O─O bonds in TBHP and generating highly oxidizable ⋅OH radicals.^[^ ^33^ ^]^ Afterwards, the S atom in the DBT molecule underwent a nucleophilic attack on the O atom of the generated ⋅OH radical to form sulfoxide (Figure 6f). The graphene‐supported MoO_2_ nanoparticle, theoretically having a higher electron density, promotes enhanced electron transfer between the catalytically active center and the oxidizing agent.^[^ ^33^ ^]^ This phenomenon contributes to the improved catalytic desulfurization capability, aligning with the experimental performance of the flash‐synthesized MoO_2_@rNC aerogel catalyst.

## 3 Conclusion

In summary, we synthesized hybrid rNC aerogels by incorporating oCNT as spacers into the nanocarbon structures, coupled with the flash Joule‐heating technique to achieve aerogels with a wide range of advantages. These include reduced carbon footprint, exceptional electrical and thermal conductivities, high thermal stabilities, substantial surface areas, well‐controlled microstructures, and superhydrophobicity. The enhanced properties of the rNC aerogel resulting from the electrothermally‐driven chemical modulation strategy were demonstrated through corresponding proof‐of‐concept studies. Notably, the superhydrophobic nature of the rNC aerogel was harnessed for separating oil/water mixtures using a custom‐made separator, achieving a significant improvement in separation flux rates compared to unreduced NC aerogel (increased by more than 72%). Moreover, the rNC aerogel exhibited efficient and durable performance under high working pressure and maintained excellent cycling capacities. The flash synthesizability was utilized to uniformly incorporate Mo‐based nano‐catalysts into the rNC aerogel, resulting in MoO_2_@rNC aerogel catalysts with outstanding catalytic oxidative desulfurization ability, achieving a DBT conversion efficiency of up to 98.9%. Additionally, our study introduced an innovative method for synthesizing MoO_3_ NPs by performing flash Joule‐heating synthesis in an air atmosphere. Overall, our study highlights the potential of flash Joule‐heating as an energy‐efficient and effective method for producing functionalized hybrid NC aerogels with versatile applications.

## 4 Experimental Section

### Hydrothermal Synthesis of Hybrid NC Aerogels

The hybrid NC aerogels were prepared using a facile hydrothermal synthesis approach with a weight ratio of 1:3 for oCNT and GO as starting materials. Specifically, 0.04 g of oCNT powders, 0.12 g of GO flakes, and 0.64 g of L‐ascorbic acid powders were mixed in 40 mL HPLC water and sonicated to obtain well‐dispersed suspensions. After that, 7.5 mL of the dispersions was transferred to a sample vial, sealed, and placed in an oven at 60 °C overnight to form the hybrid NC hydrogel. After completion, the hydrogel was washed with HPLC water to remove impurities, followed by freezing using liquid nitrogen and freeze–drying (LABCONCO) to obtain the reference NC_HT_ aerogel with dimensions of ≈1.1 cm × 1.1 cm. To obtain the NC aerogel, the NC aerogel was Joule‐heated at a current of *I* = 0.55 A for 20 min. Furthermore, ultrahigh‐temperature thermal reduction (≈3000 K) of NC aerogels by Joule‐heating at an electrical current of *I* = 10.12 A for 10, 30, and 60 s were designated as rNC_10s_ aerogel, rNC_30s_ aerogel, and rNC aerogel, respectively.

### Joule‐Heating Measurements

Joule‐heating measurements of the NC aerogels were conducted using a house‐made Joule‐heating setup in an airtight container under N_2_ atmosphere (Figure S3, Supporting Information). The NC aerogel monolith was connected to the Joule‐heating sample holder and then preconditioned at *I* = 0.55 A for 20 min to remove adsorbed water and gases. Following preconditioning, the electrical current was abruptly increased to *I* = 10.12 A to initiate the ultrahigh‐temperature thermal reduction, with the longest duration of Joule‐heating set to 60 s. Subsequently, the aerogel was allowed to cool down to ambient temperature to obtain the rNC aerogel. In addition, the ultrahigh temperature Joule‐heating periods of 10 and 30 s resulted in the formation of rNC_10s_ aerogel and rNC_30s_ aerogel, respectively, aiming to analyze their physicochemical property differences. For Joule‐heating measurements of the prepared aerogels up to 500 °C, the electrical current was incrementally increased to reach the designed temperature, following a stepwise approach. Detailed methods for recording the Joule‐heating core temperature up to around 3000 K were described in the published study. Specifically, 1D heat conduction^[^ ^36^ ^]^ and the power law^[^ ^22^ ^]^ were utilized to determine the correlation between Joule‐heating temperature and thermal conductivity. The electrical current and voltage applied to the aerogel were controlled by a portable power source (ES‐PS, 3032‐10 B). Data loggers (RS Components Ltd) were used to record the real‐time electrical current and voltage. The aerogel surface temperature and core temperature were recorded by two K grounded tip insulated probes (TJC 120 Series, TJC120‐CASS‐IM025U‐250‐HMPW‐M, RS Components Ltd, Omega UK), which were matched with data loggers (EL‐USB‐TC, Lascar Electronics) for continuous temperature readouts. The power was obtained by multiplying the recorded current with voltage, and the corresponding electrical conductivity and thermal conductivity were calculated based on studies reported elsewhere.^[^ ^36^ ^]^

### Oil–Water Separation

The NC aerogel and rNC aerogel samples were placed inside the center of a heat shrink tubing. The tubing was heated using a heat gun to facilitate the shrinking process and ensure a tight fit around the aerogel sample, thus creating a separation system. After that, 0.5 mL of oil solvents, acting as inducing agents, were dropped onto the surface of the aerogel sample. Oil/water or water/oil mixtures with varying volume ratios (ranging from 1.25:5, 2.5:5, to 5:5) were introduced into the tubing to initiate the separation process. The time elapsed from the first drop until the last drop was recorded for each sample, while the separation was ongoing.

### Preparation of Oxidative Desulfurization Catalysts

The preparation of MoO_2_@rNC aerogel catalysts and the subsequent oxidative desulfurization reactions are described below. The rNC aerogel was soaked in a solution containing 0.025 M MoO_2_(acac)_2_ in chloroform, followed by drying at room temperature. Subsequently, the MoO_2_(acac)_2_ decorated rNC aerogel was subjected to ultrahigh‐temperature Joule‐heating at *I* = 10.12 A for ≈1 s, repeated three times. This process resulted in the formation of MoO_2_@rNC aerogel catalysts. The oxidative desulfurization reactions were carried out using the same method as discussed above.

### Oxidative Desulfurization Measurements

The model fuel, containing 500 ppm S, was prepared by dissolving 1.16 g of DBT in a 400 mL hexane solution. Specifically, 5 mg of MoO_2_@rNC aerogel catalyst was added to 5 mL of the prepared model fuel, followed by 3 min bath sonication to disperse the catalyst. Then, 0.15 mL of a 70 wt.% tert‐butyl hydroperoxide (TBHP) aqueous solution was added to the catalyst‐containing mixtures. The reaction mixture was then heated to 60 °C for 12 h, under vigorous stirring. Upon the completion of the oxidative desulfurization, the solution was filtered. The liquid phase was separated and combined with 1 mL of CH_3_CN, followed by agitation at room temperature with a stirring speed of 1000 rpm for 20 min. The concentration of DBT was determined using gas chromatography (GC). For GC analysis, a sample was prepared by taking 1 mL of the supernatant phase and adding 50 µL of dodecane as an internal standard. The DBT conversion was calculated from variations in the DBT peak area observed in the GC spectrum, using a standard curve for comparison.

## Conflict of Interest

The authors declare no conflict of interest.

## Supporting information

Supporting Information

## Data Availability

The data that support the findings of this study are available from the corresponding author upon reasonable request.
