# Supplementary material for: Electrothermally‐Driven Ultrafast Chemical Modulation of Multifunctional Nanocarbon Aerogels
Source: Small. 2024 Aug 8;20(47):2404364. doi: 10.1002/smll.202404364 (PMC11579976; doi:10.1002/smll.202404364)
Supplement: Supplementary file 1 — Supporting Information [file SMLL-20-2404364-s001.docx]

*Supporting Information*

Electrothermally-Driven Ultrafast Chemical Modulation of Multifunctional Nanocarbon Aerogels

Dong Xia, Qun Li*, Jamie Mannering, Yi Qin, Heng Li, Yifei Xu*, Ashiq Ahamed, Wenyu Zhou, Alexander Kulak and Peng Huang*

Experimental Procedures

*Chemicals:* Graphene oxide (GO) sheets were purchased from William Blythe Limited, UK. Carboxylic-acid-functionalized multi-walled carbon nanotubes (oCNT), dibenzothiophene (DBT), L-ascorbic acid, rhodamine B, methylene blue (MB), and bis(acetylacetonato)dioxomolybdenum(VI) (MoO_2_(acac)_2_) were obtained from Sigma-Aldrich. HPLC water, chloroform (CHCl_3_), dichloromethane (CH_2_Cl_2_), dichlorobenzene (C_6_H_4_Cl_2_), tert-Butyl hydroperoxide (TBHP), hexane, dodecane and acetonitrile (CH_3_CN) were sourced from Fisher Scientific, UK. Heat shrinkage tubing was supplied by 3M company, UK. All chemicals were used as received.

*Materials characterizations:* X-ray diffraction (XRD) measurements were tested on a Bruker D2 Phaser Diffractometer. Scanning electron microscopy (SEM) analysis was performed on a Nova NanoSEM 450 with an accelerating voltage of 3 kV. Energy dispersive X-ray spectroscopy (EDX) map was collected from the same SEM instrument at an accelerating voltage of 15 kV. Contact angles of aerogel materials were tested using a drop-shape analysis device (OCA 25, Dataphysics UK). Thermogravimetric analysis (TGA) was performed on a TGA Q600 model (TA Instruments) using a 10 ^o^C/min ramping rate from 20 to 850 ^o^C in air atmosphere. Raman spectroscopy analysis was conducted on a Renishaw InVia Raman microscope, with an excitation laser wavelength of 532 nm between 400 and 4000 cm^-1^. Raman mapping was performed on the same instrument with an excitation laser wavelength of 785 nm ranging from 1,000 and 2,300 cm^-1^. Thermal images were taken using a Fluke TiR1 thermal camera, and the obtained pictures were analyzed *via* Fluke Connect software. X-ray photoelectron spectroscopy (XPS) was performed using a Thermo Fisher Scientific K-Alpha^+^ X-ray photoelectron spectrometer; high-resolution scans of elemental peaks were collected at a pass energy of 30 eV and a step size of 0.1 eV. The binding energies were referenced to the C 1s peak of adventitious carbon at 284.8 eV. Brunauer-Emmett-Teller (BET) surface area results were measured by a Micromeritics TriStar 3000 instrument. The specimens were degassed in nitrogen gas at 110 ^o^C for 3 hours prior to analysis, and nitrogen adsorption/desorption isotherms were measured at 77 K. Transmission electron microscopy (TEM) was measured on an electron microscope (Tecnai F30, FEI), at an accelerating voltage of 300 kV. The aerogel samples were dispersed in ethanol, followed by drop-casting onto a copper grid.

Life cycle analysis (LCA) was performed following the guidelines outlined in ISO 14040 and 14044 standards. The primary objective of this study was to conduct a comparative analysis of the Joule heating method against two conventional oven-based heating methods, all of which are commonly employed in the synthesis of aerogels. The study’s scope extended to a cradle-to-gate assessment, encompassing an analysis of the entire life cycle of aerogel production, including the raw materials and the synthesis process, as represented in Figure 1. The functional unit was defined as the synthesis of 1 cm^3^ of aerogel. The life cycle inventory was developed based on the experimental research conducted in this study and literature sources related to conventional heating methods. The IPCC GWP 100 method was employed for the assessment. The SimaPro software (V9.4, PRé Sustainability B.V.) equipped with the ecoinvent database (V3.9) was used for the LCA study.

*DFT calculations:* DFT calculations were performed using the Vienna Ab Initio Simulation Package (VASP)[37, 38] with generalized gradient approximations. The Perdew-Burke-Ernzerhof functional was used for the exchange correlation.[39] Spin-polarized and Grimme dispersion correction (DFT-D3)[40] were considered in all calculations to reveal the influence of *van der* Waals interaction. The projector-augmented wave method was utilized to model the valence-electron configuration of C, Mo and O with an energy cut-off of 520 eV.[41] The MoO_2_ (110) surface derives from fully relaxed MoO_2_ bulk with a space group of P2_1_/c, the six-layered slabs model was adopted for the modelling of the most stable terminator.[42] The periodic boundary conditions were applied to simulate all the structures. At least a 15 Å vacuum layer was used to hinder the interaction of stacked slabs. The *k*-point grids adopt the 2×2×1 *k*-point meshes for Brillouin zone sampling.[39] For pure graphene, the 12×12×4 *k*-point meshes were used for precise calculations of the densities of states (DOS).

For the MoO_2_(110)@graphene, based on lattice parameters of graphene and MoO_2_(110) surface, which consists of a graphene supercell (5 × 3) and MoO_2_(110) surface (2 × 1) were constructed using as small as possible lattice mismatch of around 8 %. If the crystal lattice mismatch is too significant, the interface becomes unstable due to the high interfacial stress. The relaxed structures of MoO_2_(110)@graphene are depicted in Figure S25.

The convergence criteria were set to less than 0.02 eV/Å for the force and 10^-5^ eV/atom for the energy, respectively. The charge density difference was also employed to qualify the charge transfer at the interface of MoO_2_(110)@graphene. All structure schematic diagrams were visualized by VESTA.[43]


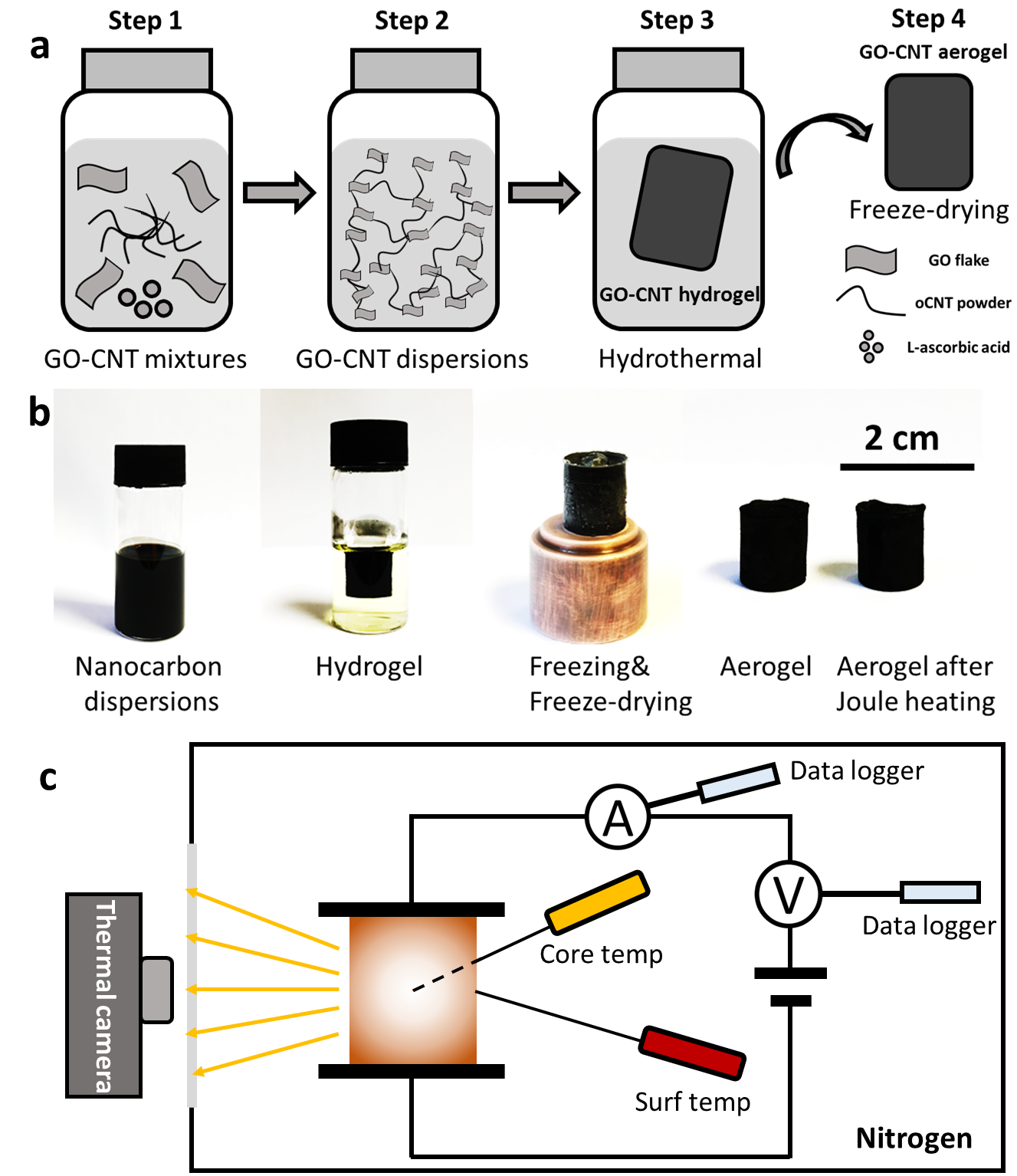


**Figure S1.** (a) Schematics description of the hydrothermal approach to synthesize the hybrid NC aerogels.


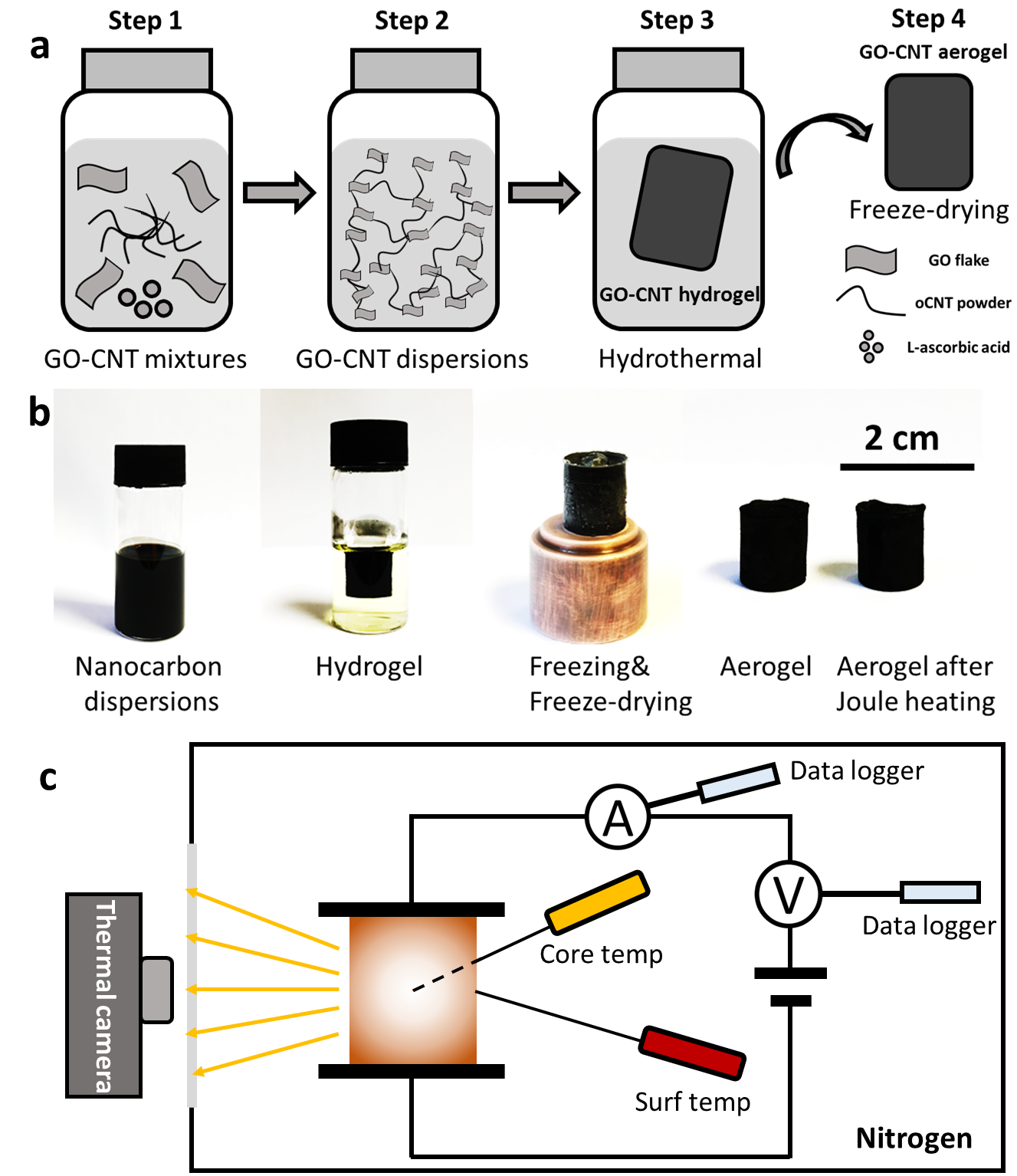


**Figure S2.** Corresponding digital photos of each synthesizing step.

Graphene oxide (GO, 3 mg⋅cm^‑3^) and acid oxidized multiwalled carbon nanotubes (oCNTs, 1 mg⋅cm^-3^) are exfoliated and dispersed within an aqueous solution of L‑ascorbic acid (16 mg⋅cm^-3^) and subjected to an elevated temperature (60 °C for 12 h) within a gas-tight vial. The nanocarbons are partially reduced by L-ascorbic acid during this process and spontaneously self-assemble into a porous nanocarbon network through non-covalent van der Waals interactions, enabling the formation of a stable hybrid NC hydrogel network. After the freeze-drying step, a free-standing and structurally intact NC aerogel, serving as the Joule-heating aerogel precursor, is obtained (Figure S2). The utilization of oCNTs (avg. length 2 µm and width 18 nm) inhibits extensive GO restacking during partial chemical reduction through a steric bridging function (similar to graphene intercalation) while the GO itself functions as the primary network former (GO lateral dimensions 0.8 × 0.8 µm). The efficacy of oCNTs in mitigating GO restacking is determined through a comparison of the powder X-ray diffraction (XRD) pattern (002 diffraction peak analysis) of the dried hybrid NC aerogel with a GO aerogel fabricated under the same conditions but without oCNTs. The (002) XRD peak for the hybrid oNC aerogel appears broad, and Scherrer analysis determines fewer sheets (NC aerogel 5 layers, GO aerogel 8 layers) per crystallographic domain, indicating less GO stacking during hybrid assembly (Figure S3 and Table S1).


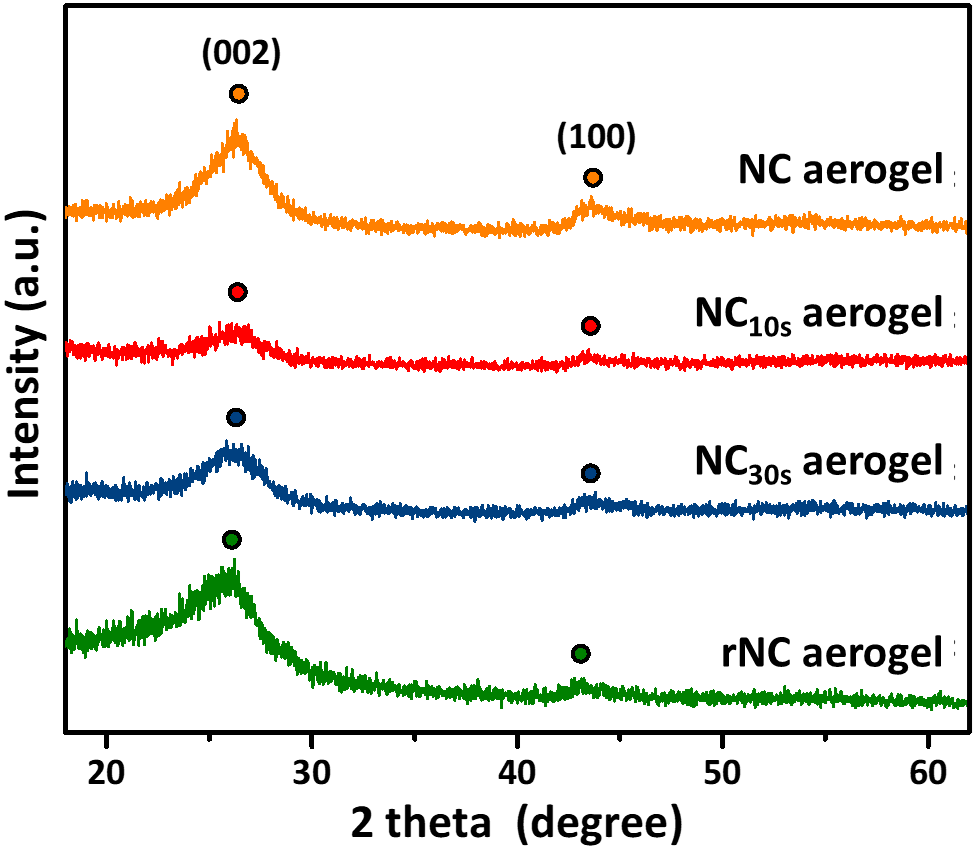


**Figure S3**. XRD patterns of NC aerogel, NC_10s_ aerogel, NC_30s_ aerogel, and rNC aerogel. The pronounced characteristic (002) peak is more crystallized and sharper with the arising Joule-heating time.

**Table S1.** Physical parameters of nanocarbon aerogels derived from the diffraction peak

| Aerogel name | Peak position 2θ_(002)_ (°) | Full Width at Half Maximum (°) | D_p_  (nm) | *d*-spacing (nm) | Layer  (n) |
| --- | --- | --- | --- | --- | --- |
| NC aerogel | 25.49 | 5.89 | 1.45 | 0.35 | 5 |
| rNC_10s_ raerogel | 25.88 | 4.17 | 2.04 | 0.34 | 7 |
| NC_30s_ aerogel | 25.90 | 4.38 | 1.95 | 0.34 | 7 |
| rNC aerogel | 26.18 | 3.60 | 2.37 | 0.35 | 8 |


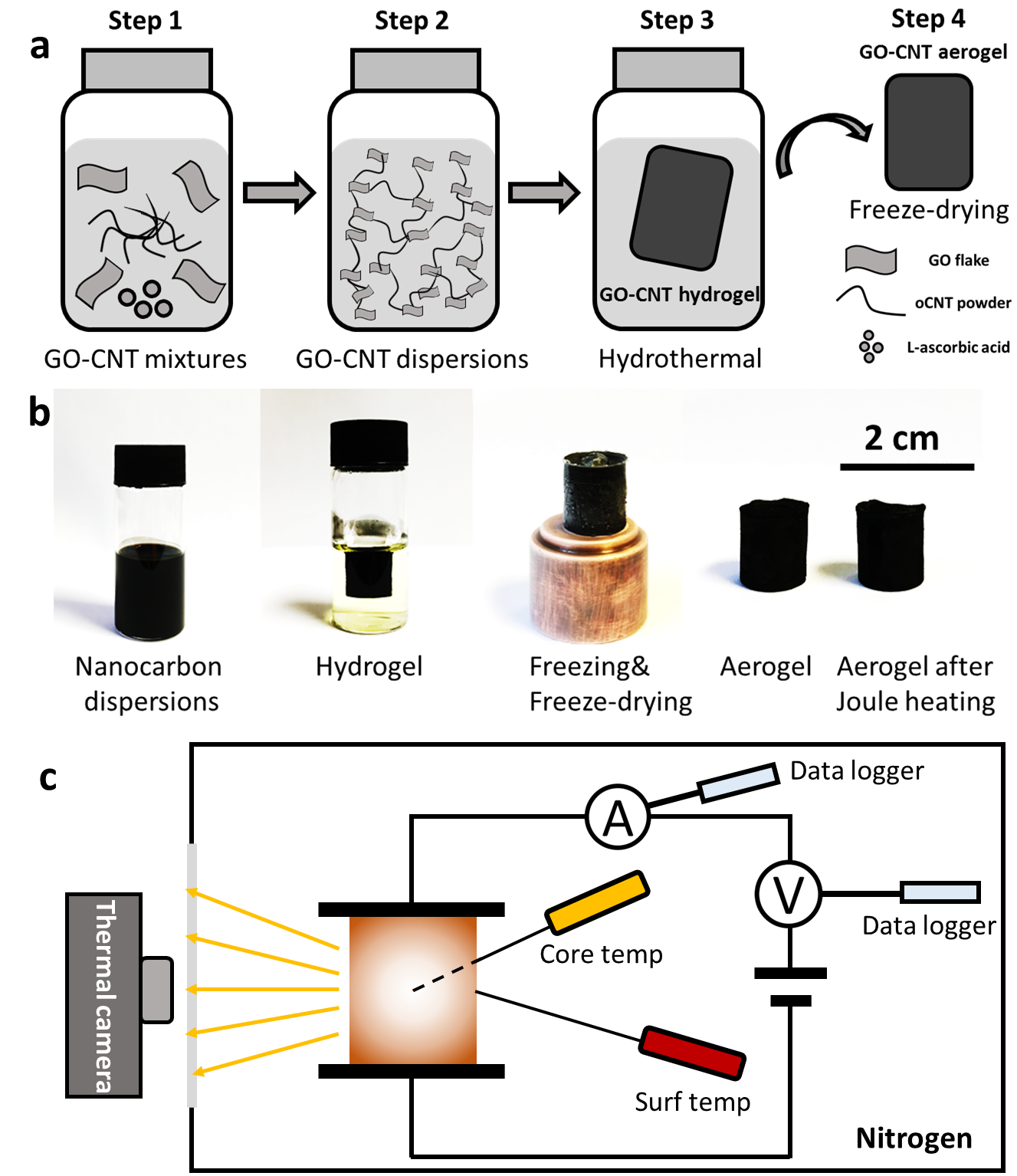


**Figure S4.** Detailed set-up for Joule-heating, illustrating the electrical-thermal experiments and data recording process.

**Estimation of Joule-Heating Temperatures for the rNC Aerogels**

Due to heat conduction and thermal losses from the center to the surface of the aerogel, the temperature of the aerogel during Joule-heating was estimated using a simplified one-dimensional heat conduction model (Equation S1).^[1]^ The model allows for the determination of the surface temperature (*T_surf_*) and core temperature (*T_core_*) of the aerogel at a fixed electrical power input (*q*), based on the thermal conductivity (*k*) and the distance (*r*) between the core and surface position of the aerogel. The fitting parameter (*C_rad_*) is obtained using Equation S2. The detailed process for recording the core and surface temperature during Joule-heating is illustrated in Fig. S5a, and the temperature gradient is shown in Figure S5b.

$T_{core}=T_{surf}+\frac{q}{4k}r^{2}$ Equation (S1)

$C_{rad}=\frac{q}{4k}r^{2}$ Equation (S2)


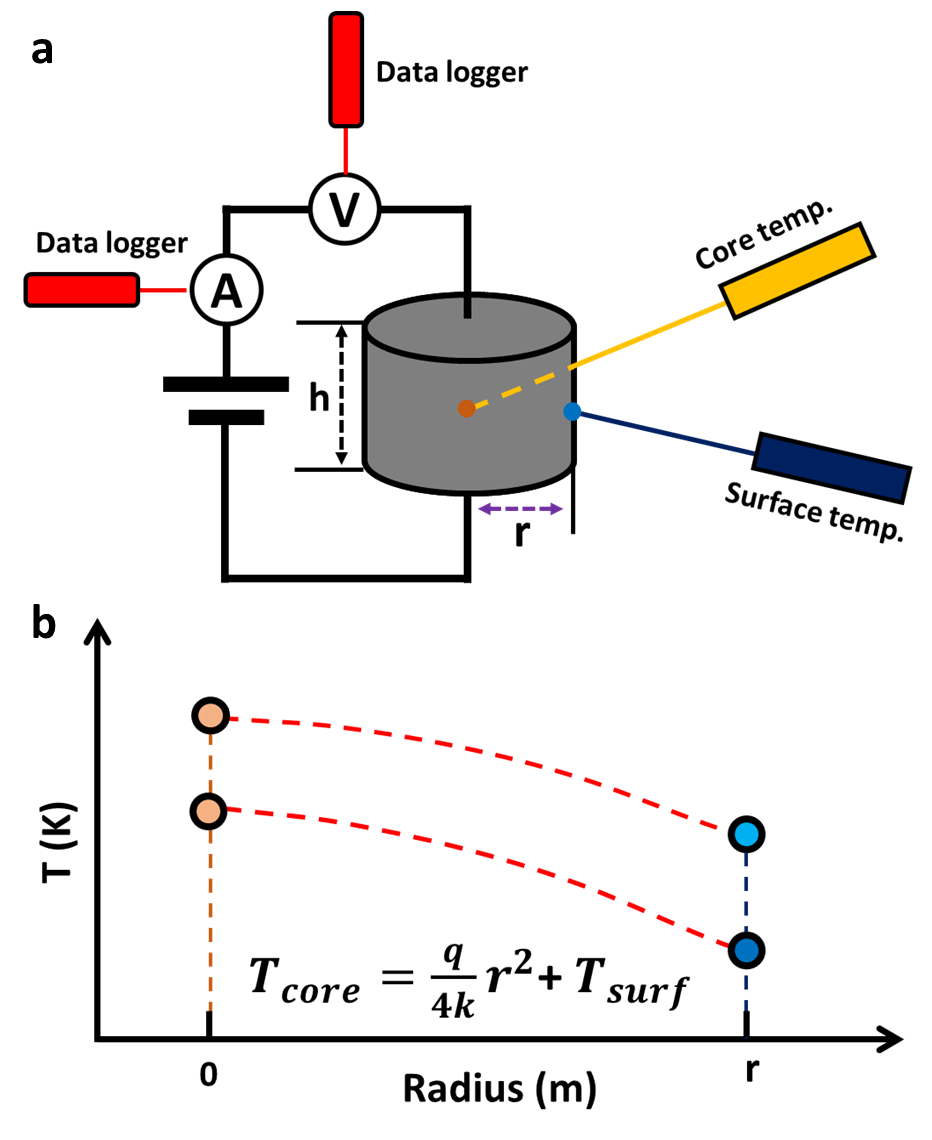


**Figure S5.** (a) Schematics depicting the recording of core temperature (for Joule-heating core temperature < 1200 ^o^C) and surface temperature of the rNC aerogel using thermocouples. (b) Relationship between thermal conductivity and core temperature of the rNC aerogel using the simplified one-dimensional heat conduction model (Equation S1).

Because of the extremely high temperature at the center of the aerogel, direct measurement of the core temperature is not possible using methods such as a thermocouple (which melts at ~1200 ^o^C), a high-temperature thermal camera, or optical fiber spectroscopy (which only provides surface temperature readings). Therefore, the estimation of the ultrahigh core temperature was done using a combination of a thermocouple, thermal camera, and the power law fitting method (Equation S3, Figure S5, and Figure S6), in conjunction with Equation S1. More detailed information about the power law fitting method can be found in a previously reported study.^[2]^

$k_{HighTemp}=k_{KnownT}\left( \frac{T_{HighTemp}}{T_{Known}} \right)^{n}$ Equation (S3)

where *T*_HighTemp_ is the core temperature, *k*_HighTemp_ represents the thermal conductivity of the core temperature, *k_KnownT_* is the measured thermal conductivity at the inflection temperature point, *T_KnownT_* is the measured temperature at the inflection temperature point, and *n* is the power law fitting parameter.

**
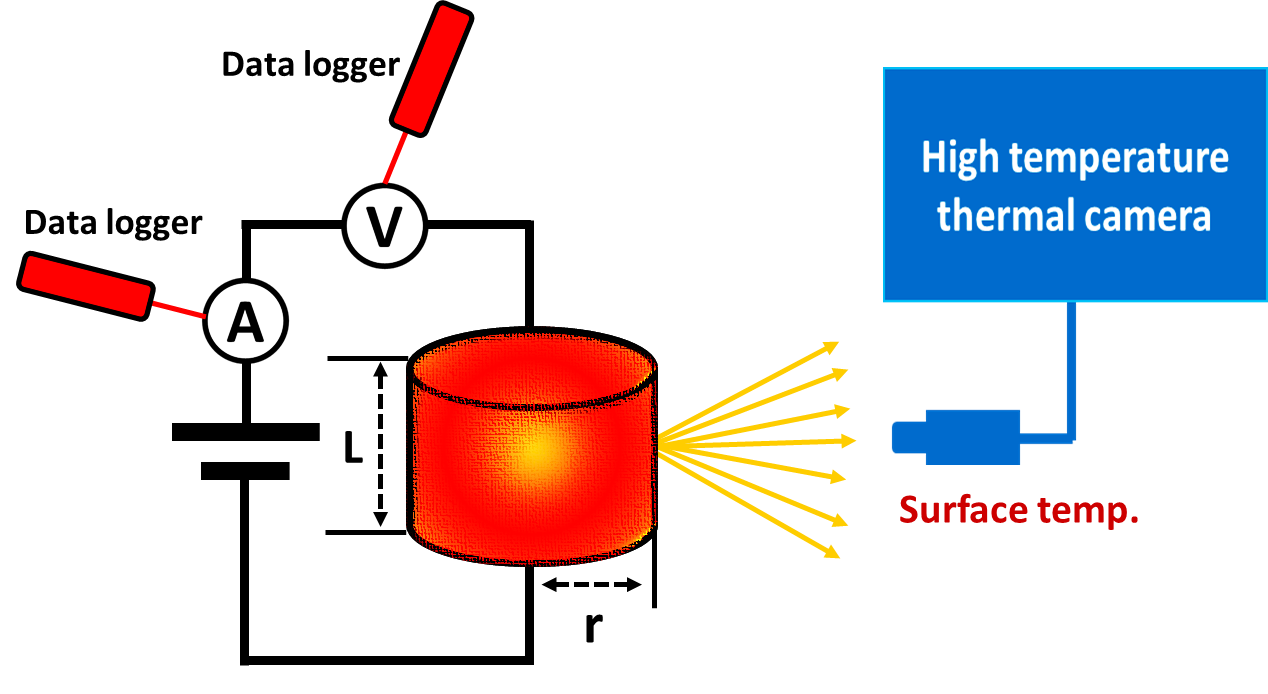
**

**Figure S6.** Schematics illustrating the recording of ultrahigh surface temperature (> 1200 ^o^C) using a thermal camera.

The temperature difference between the core temperature and the surface temperature can be attributed to heat conduction, as described above (Figure S7a). The plot of thermal conductivity as a function of surface temperature (Figure S7b) shows a reflection point at 615 K, while the relationship for the core temperature is depicted in Figure S8c. The obtained data was fitted using a power law expression to determine the *n* value (n = -0.454), and the thermal conductivity is 1.196 W⋅m^-1^⋅K^-1^. Subsequently, the power law equation was used to convert Equation S4 into a format that allows the estimation of the ultrahigh surface temperature using the thermocouple and thermal camera. This provides a correlation between surface temperature and thermal conductivity, as shown in Figure S7b. By applying Equation S1, the core temperature can be calculated, and its correlation with the thermal conductivity is demonstrated in Figure S7c.

$k_{HighTemp}=1.196\times\left( \frac{T_{HighTemp}}{615} \right)^{-0.454}$ Equation (S4)

**
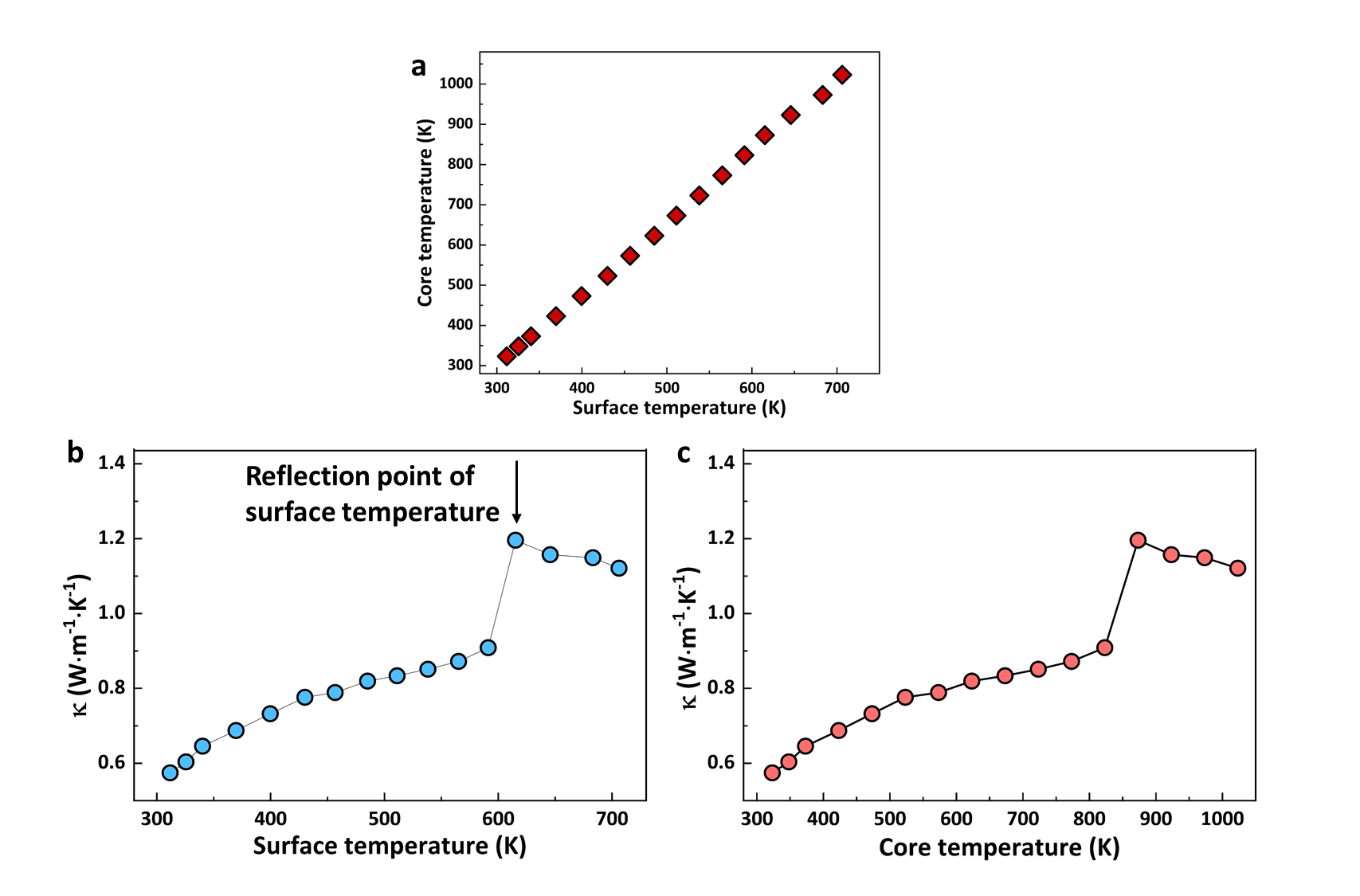
**

**Figure S7.** (a) Temperature difference between the core temperature and surface temperature of the rNC aerogel during Joule-heating. (b) Thermal conductivity of the rNC aerogel versus the Joule-heating surface temperature, showing a reflection point at 615 K. (c) Thermal conductivity of the rNC aerogel versus the Joule-heating core temperature.

The correlation between surface/core temperatures and thermal conductivity of the rCN aerogel is displayed in Figure S8, indicating that at a current of I=10.12 A, the rNC aerogel reaches temperatures of ~3,000 K. Importantly, this fitting result also demonstrates a high linear correlation between the aerogel core temperature and power input, emphasizing its reliability. In addition, this proposed method for obtaining the ultrahigh core temperature of the rNC aerogel was further evaluated at a surface temperature of 706 K, with an *n* value of -1 (where n= -1 represents the classic Umklapp scattering). However, the resulting core temperature at I=10.12 A was 3,753 K, which was inaccurate in this context.


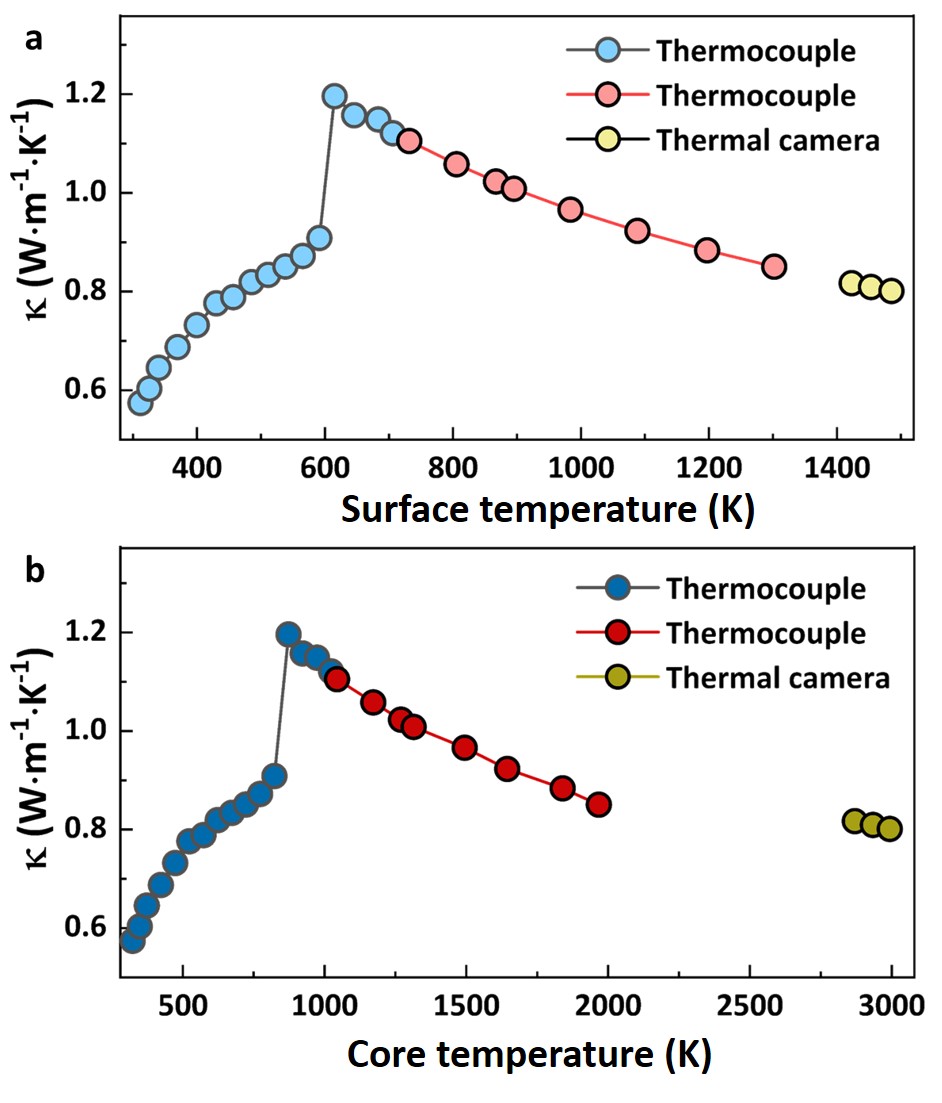


**Figure S8.** (a) Correlation between the Joule-heating surface temperature and thermal conductivity of the rNC aerogel. (b) Correlation between the Joule-heating core temperature and thermal conductivity of the rNC aerogel.


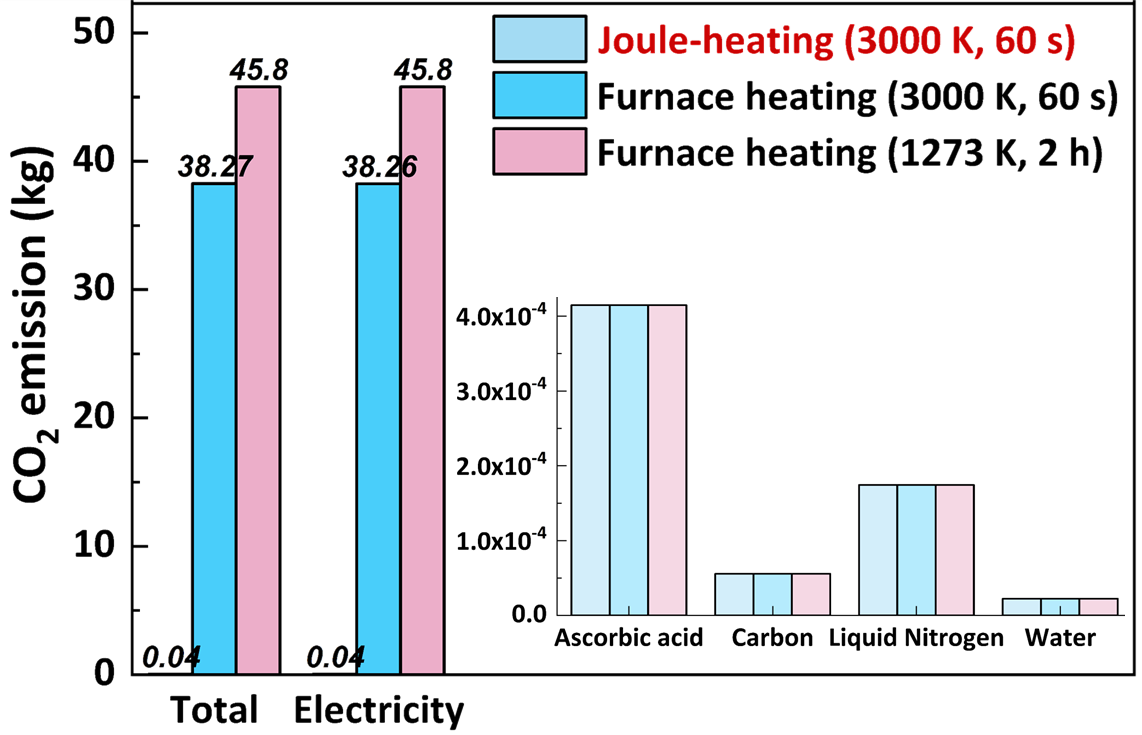


**Figure S9.** Global warming potential of Joule-heating versus conventional furnace heating for aerogel annealing.

**Table S2.** Life cycle inventory for the synthesis of 1 cm^3^ of aerogel via different methods.[44, 45]

| Materials (g) | Joule Heating Method | Conventional Heating Method 1 | Conventional Heating Method 2 |
| --- | --- | --- | --- |
| oCNT | 0.0075 | 0.0075 | 0.0075 |
| GO | 0.0225 | 0.0225 | 0.0225 |
| L-ascorbic acid | 0.12 | 0.12 | 0.12 |
| Ultrapure Water | 7.5 | 7.5 | 7.5 |
| Liquid N_2_ | 0.75 | 0.75 | 0.75 |
|  |  |  |  |
| Electricity (kWh) |  |  |  |
| Oven 60°C, 12h | 0.00288 | 0.00288 | 0.00288 |
| Freeze drying, 24h | 0.1104 | 0.1104 | 0.1104 |
| Pre-treatment (heat, 150°C, 20 min) | 0.001167 | 14 | 14 |
| UHT Thermal Reduction | 0.0025 |  |  |
| Thermal Reduction | -- | 100 | 122.44 |

**Note:** The washing step for all three scenarios was excluded due to a lack of appropriate data.

Table S2 shows the data inventory for all the steps involved in the three methods. The data reflect the synthesis of 1 cm^3^ of aerogel, derived from 7.5 mL of the mixture. Additionally, the inventory was extrapolated from the full capacity of the respective equipment used in the synthesis. For example, the capacity of the oven was 19 L, which was extrapolated to 7.5 mL as required per functional unit in this LCA.

**Table S3.** CO_2_e emission values for the synthesis of 1 cm^3^ of aerogel via different methods

| Process (kgCO_2_e) | Joule Heating Method | Conventional Heating Method 1 | Conventional Heating Method 2 |
| --- | --- | --- | --- |
| Ascorbic acid | 4.15 × 10^-4^ | 4.15 × 10^-4^ | 4.15 × 10^-4^ |
| Carbon | 5.57 × 10^-5^ | 5.57 × 10^-5^ | 5.57 × 10^-5^ |
| Electricity | 3.92 × 10^-2^ | 38.28 | 45.80 |
| Liquid N_2_ | 1.74 × 10^-4^ | 1.74 × 10^-4^ | 1.74 × 10^-4^ |
| Water | 2.25 × 10^-5^ | 2.25 × 10^-5^ | 2.25 × 10^-5^ |
| Total | 3.99 × 10^-2^ | 38.28 | 45.80 |

The CO_2_ emission equivalents are generated by inputting the relevant parameters from Table S2 into the software model. The LCA details of the derived emissions for ascorbic acid, carbon, liquid nitrogen, and water are now included in Table S3. The outcome from the LCA software is presented in Figure S9.


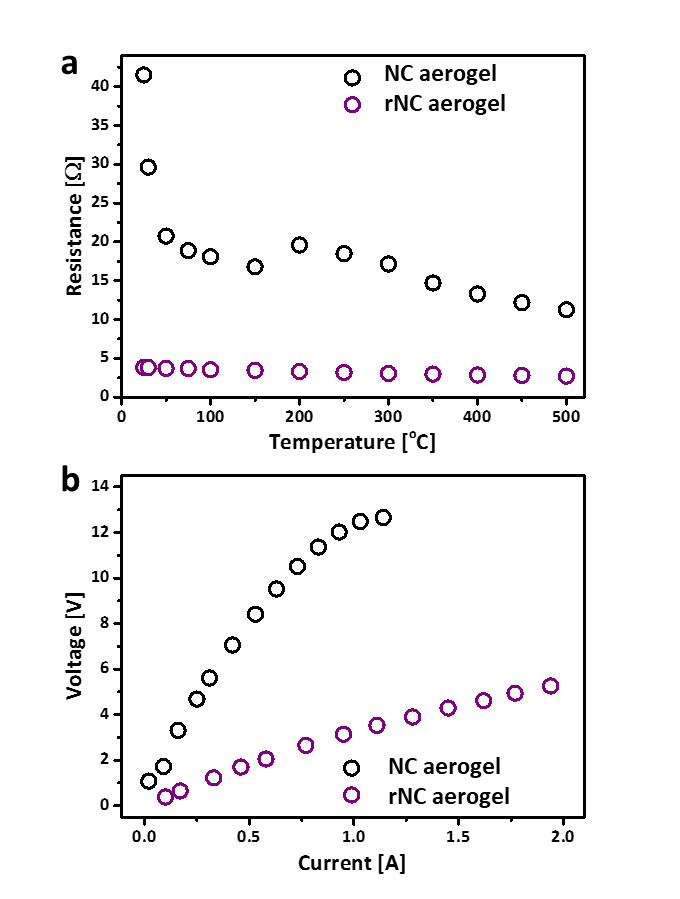


**Figure S10.** (a) Resistance versus Joule-heating temperature of NC aerogel and rNC aerogel. (b) Electrical voltage and current relationships of NC aerogel and rNC aerogel.


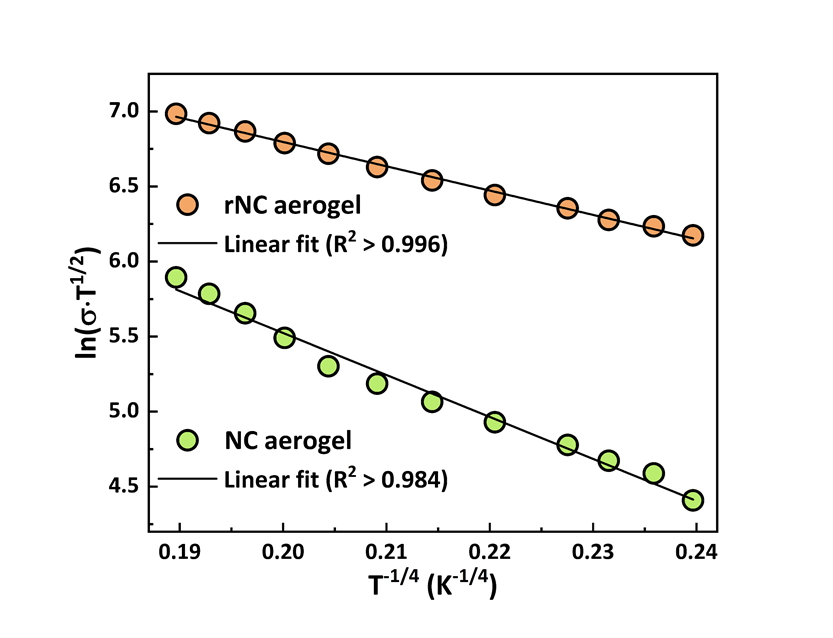


**Figure S11.** Fit of 3D variable hopping model applied to NC aerogel and rNC aerogel.


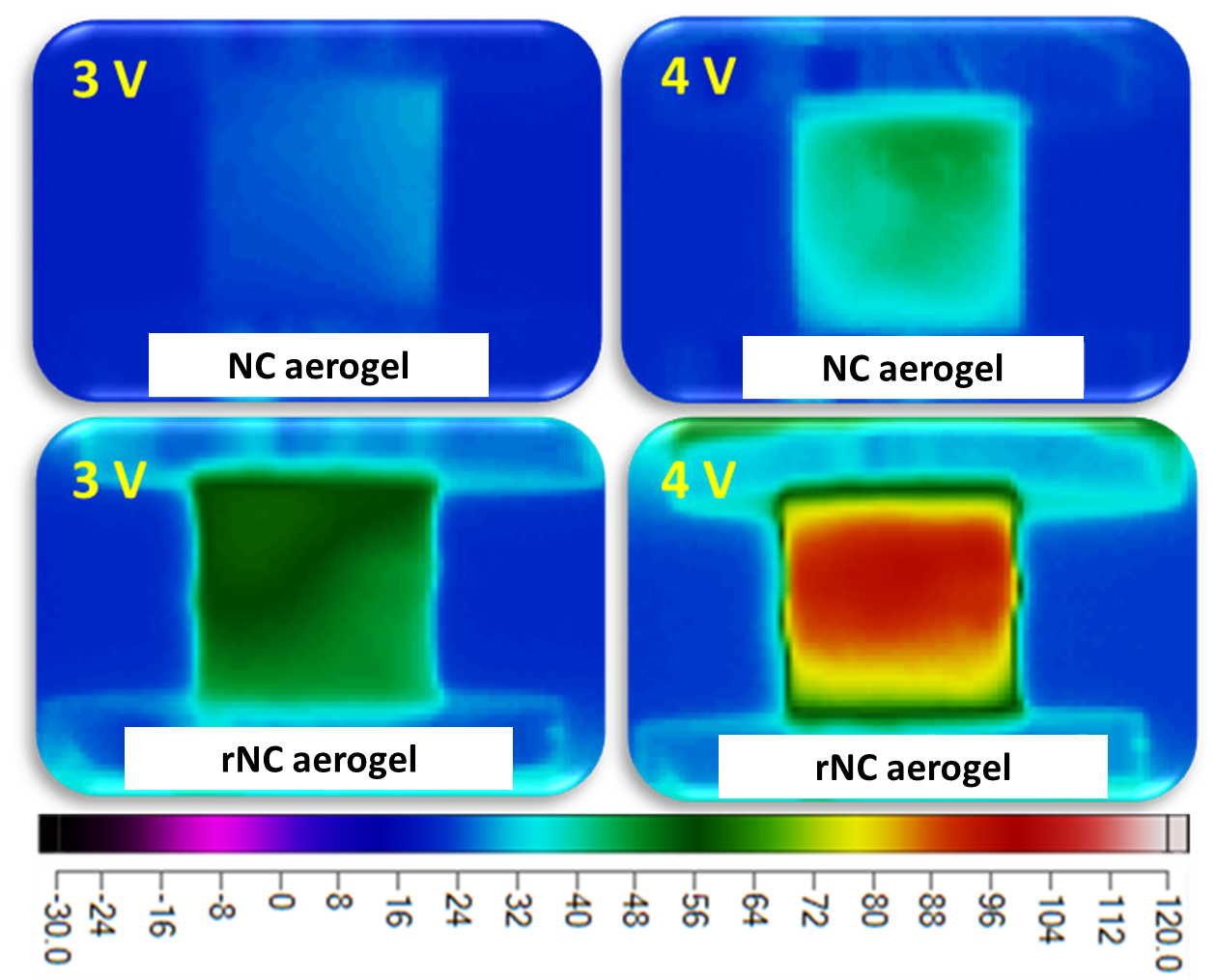


**Figure S12.** Thermal images of NC aerogel and rNC aerogel at different voltages. The results demonstrate that the rNC aerogel treated with ultrahigh temperature Joule-heating exhibits much lower resistance than the unreduced NC aerogel, indicating higher energy efficiency.


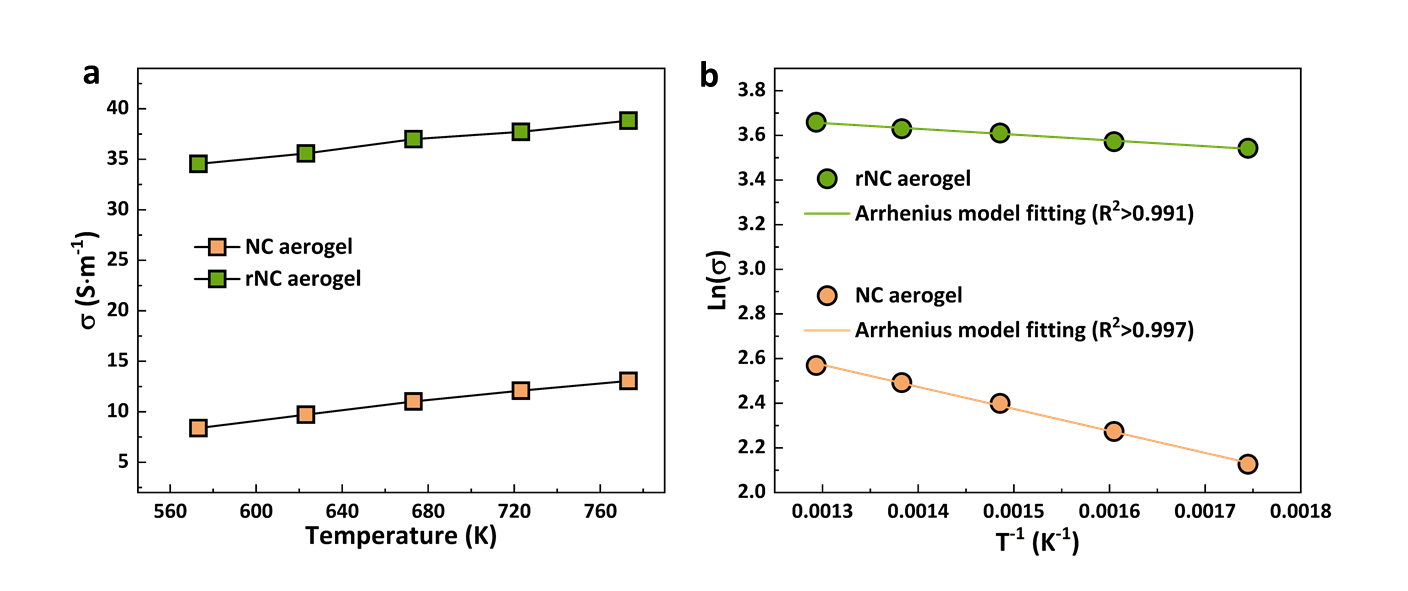


**Figure S13.** (a) Electrical conductivity as a function of Joule-heating temperature of NC aerogel and rNC aerogel. (b) Arrhenius model fitting of NC aerogel and rNC aerogel.


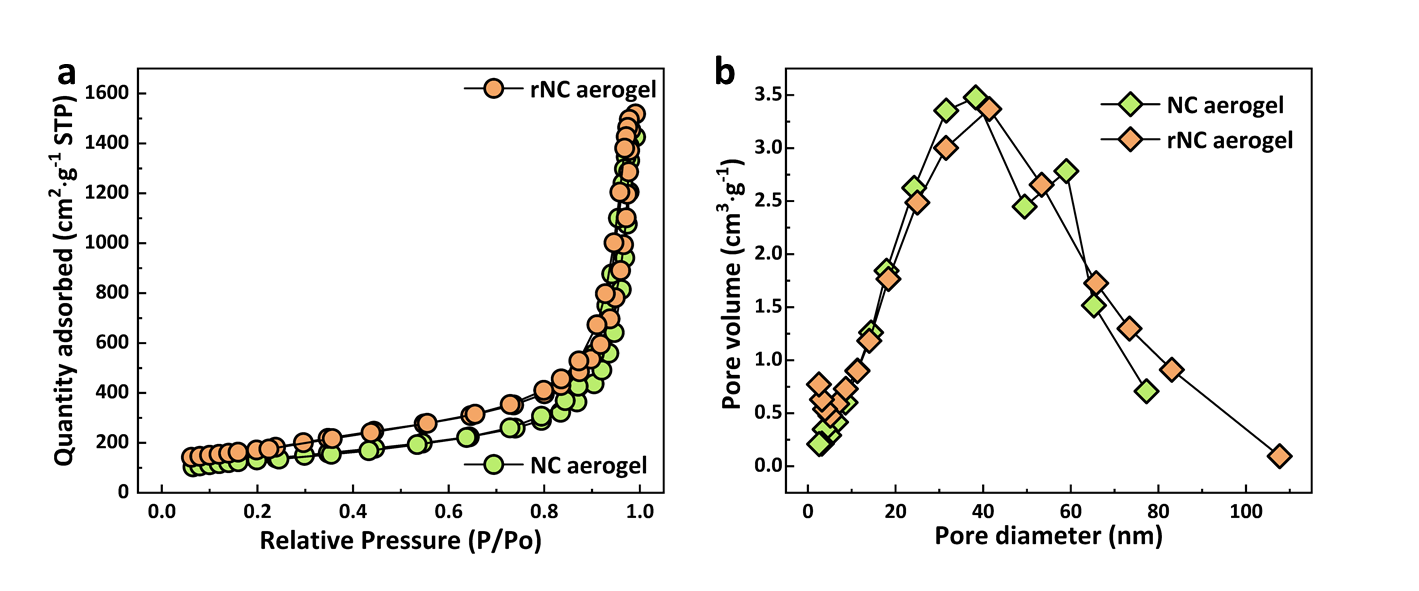


**Figure S14.** Nitrogen adsorption-desorption curves and pore-size distributions of NC aerogel and rNC aerogel.


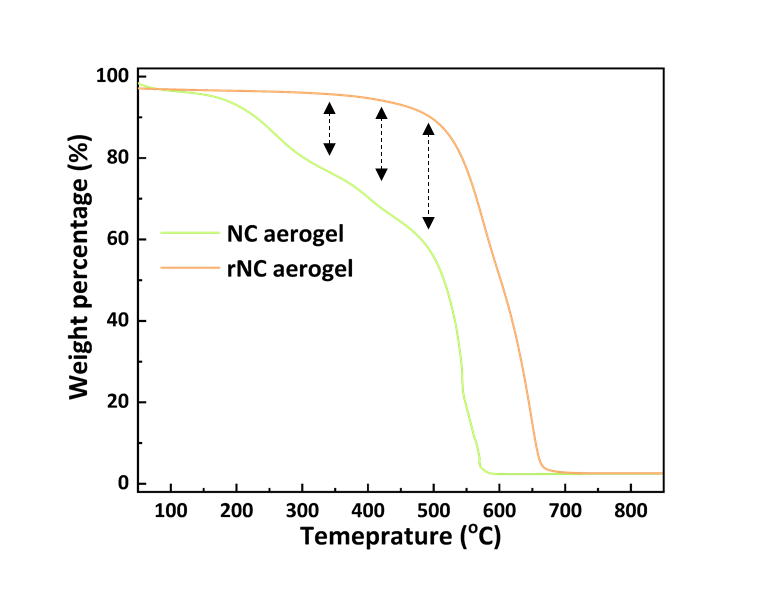


**Figure S15.** TGA curves of NC aerogel and rNC aerogel.


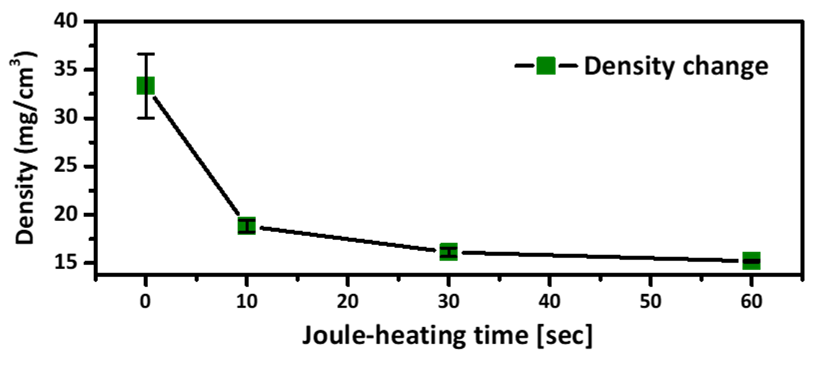


**Figure S16.** Aerogel density as a function of Joule-heating duration at I=10.12 A.


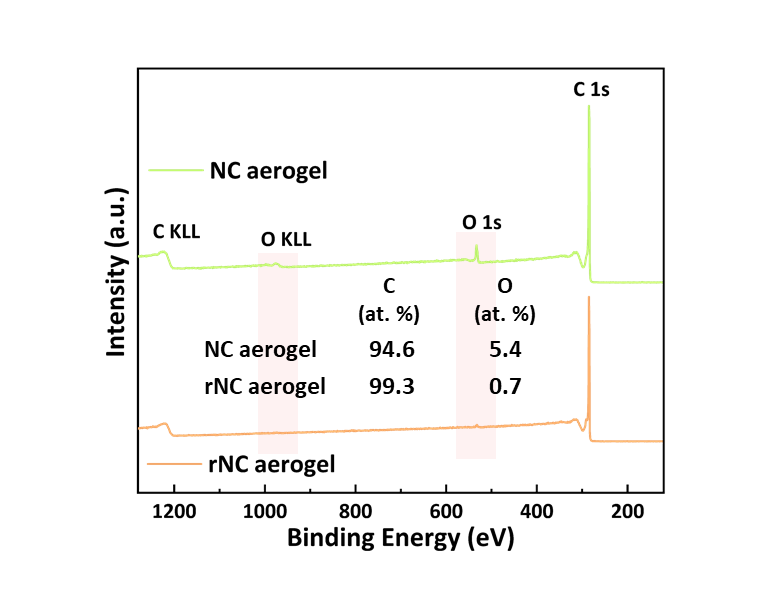


**Figure S17.** XPS full survey spectra of NC aerogel and rNC aerogel.


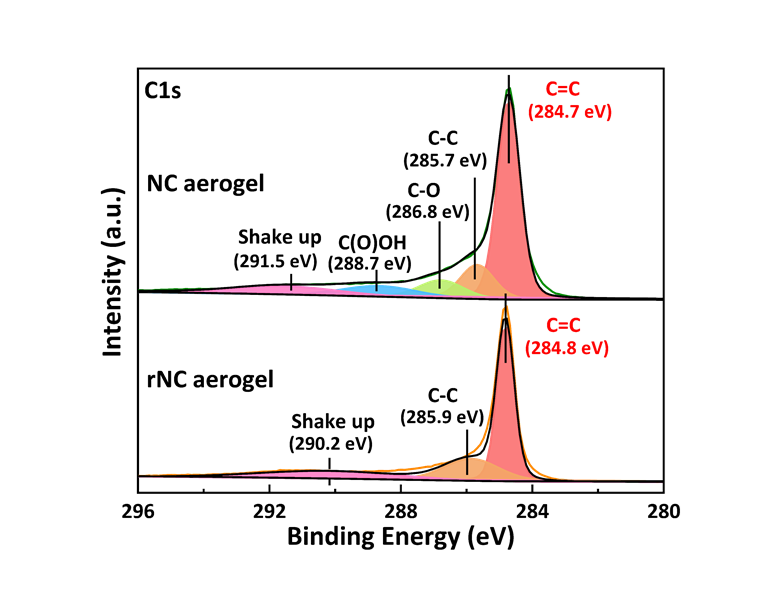


**Figure S18.** High-resolution XPS C1s region spectra of NC aerogel and rNC aerogel.


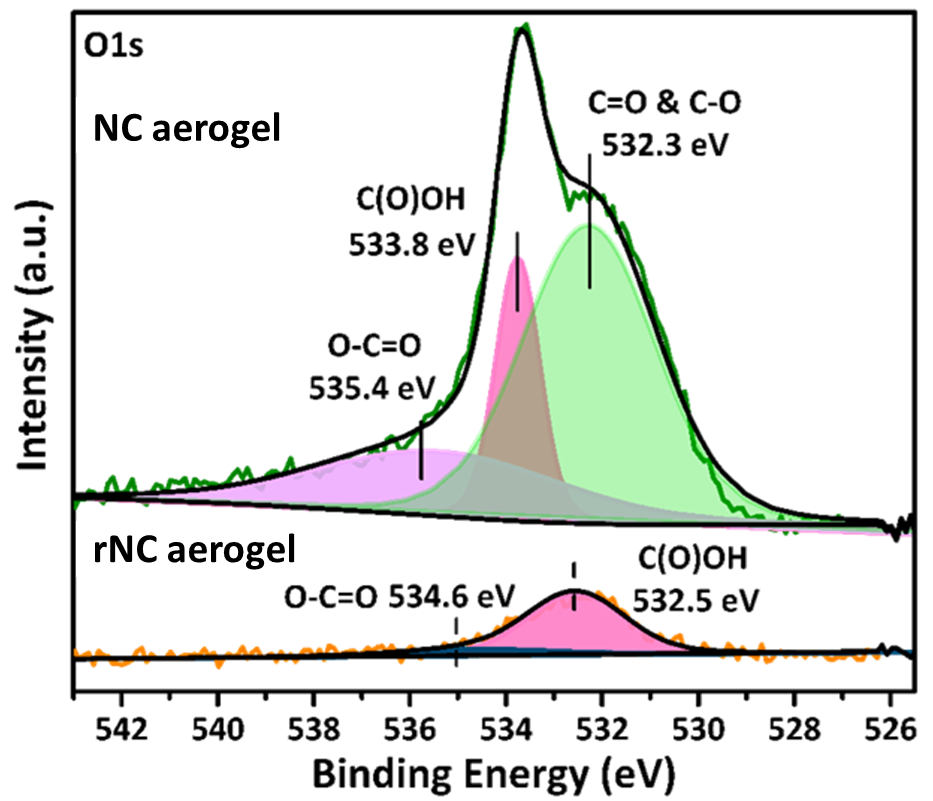


**Figure S19.** XPS O1s region of the NC aerogel and rNC aerogel.


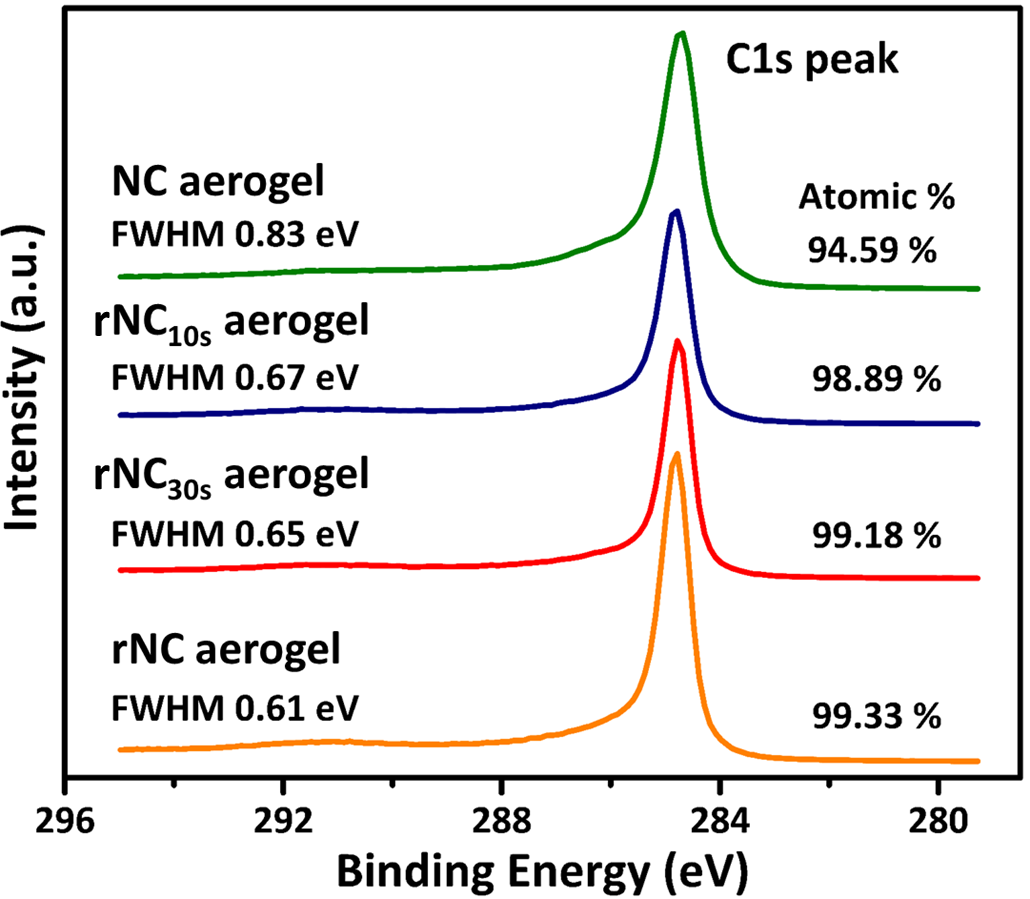


**Figure S20.** High-resolution XPS C1s peak of NC aerogel, rNC_10s_ aerogel, rNC_30s_ aerogel, and rNC aerogel. The full width at half maximum (FWHM) is decreased substantially meaning more crystallized structures. Additionally, the carbon compositions are dominated gradually, confirming the successful removal of residual oxygenic functional groups.

In the Raman spectrum of graphene, the D band is known as the disorder or defect band and represents a ring-breathing mode from sp^2^ carbon rings. The G band is a primary in-plane vibrational mode that represents the planar configuration of sp^2^ bonded carbon that constitutes graphene. The 2D band is caused by a second-order overtone of a different in-plane vibration. High-quality graphene and its derivatives (e.g., GO, rGO) typically show a stronger 2D band, indicating higher graphitic quality. The emergence of a strong 2D peak suggests increased graphitization. Changes in the G peak, particularly its intensification, indicate the restoration of more planar configuration sp^2^ bonded carbon in the nanocarbons, signifying improved graphiticity. A commonly used indicator to assess the graphiticity of defective graphene oxide (GO) materials is the I_D_/I_G_ ratio. A smaller I_D_/I_G_ value indicates higher graphiticity. The results showed that the resultant rNC aerogel exhibits the smallest I_D_/I_G_ value (Figures 3a-3b), indicative of high graphiticity.


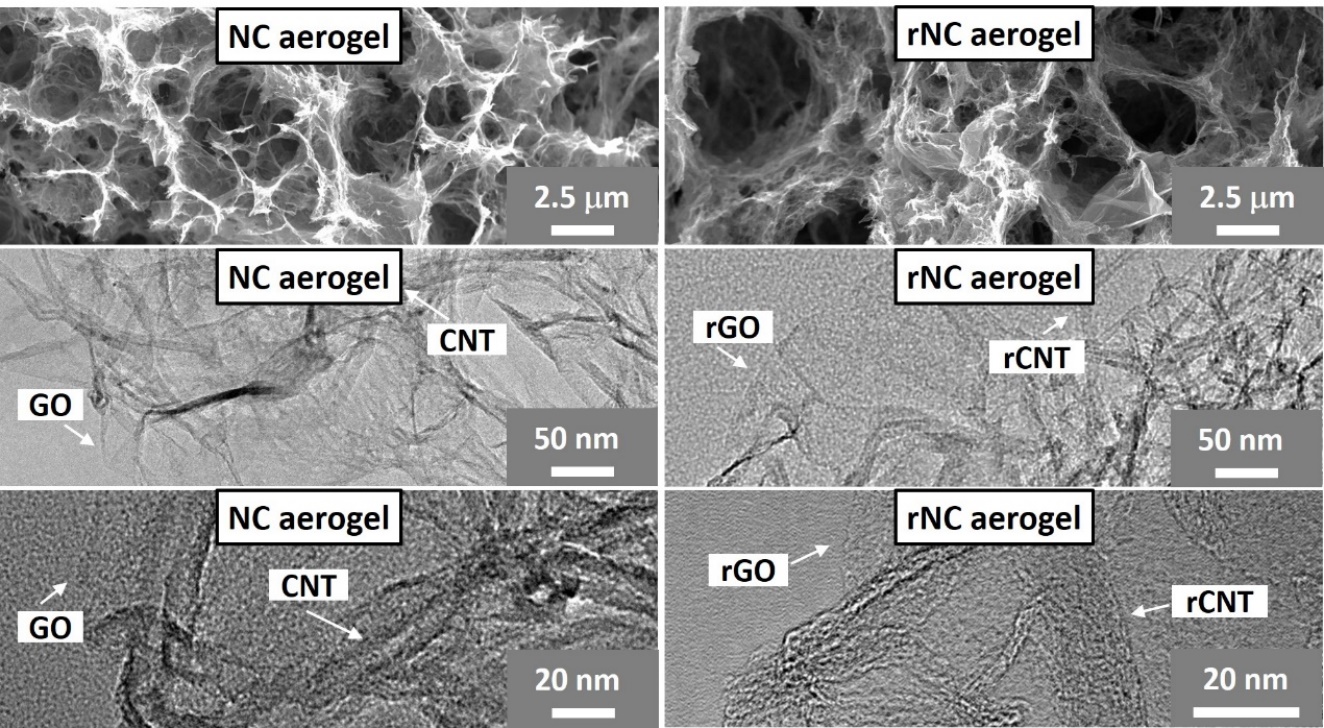


**Figure S21.** SEM images of NC aerogel (a) and rNC aerogel (b). TEM images of NC aerogel (c, e) and rNC aerogel (d, f) at different magnifications.


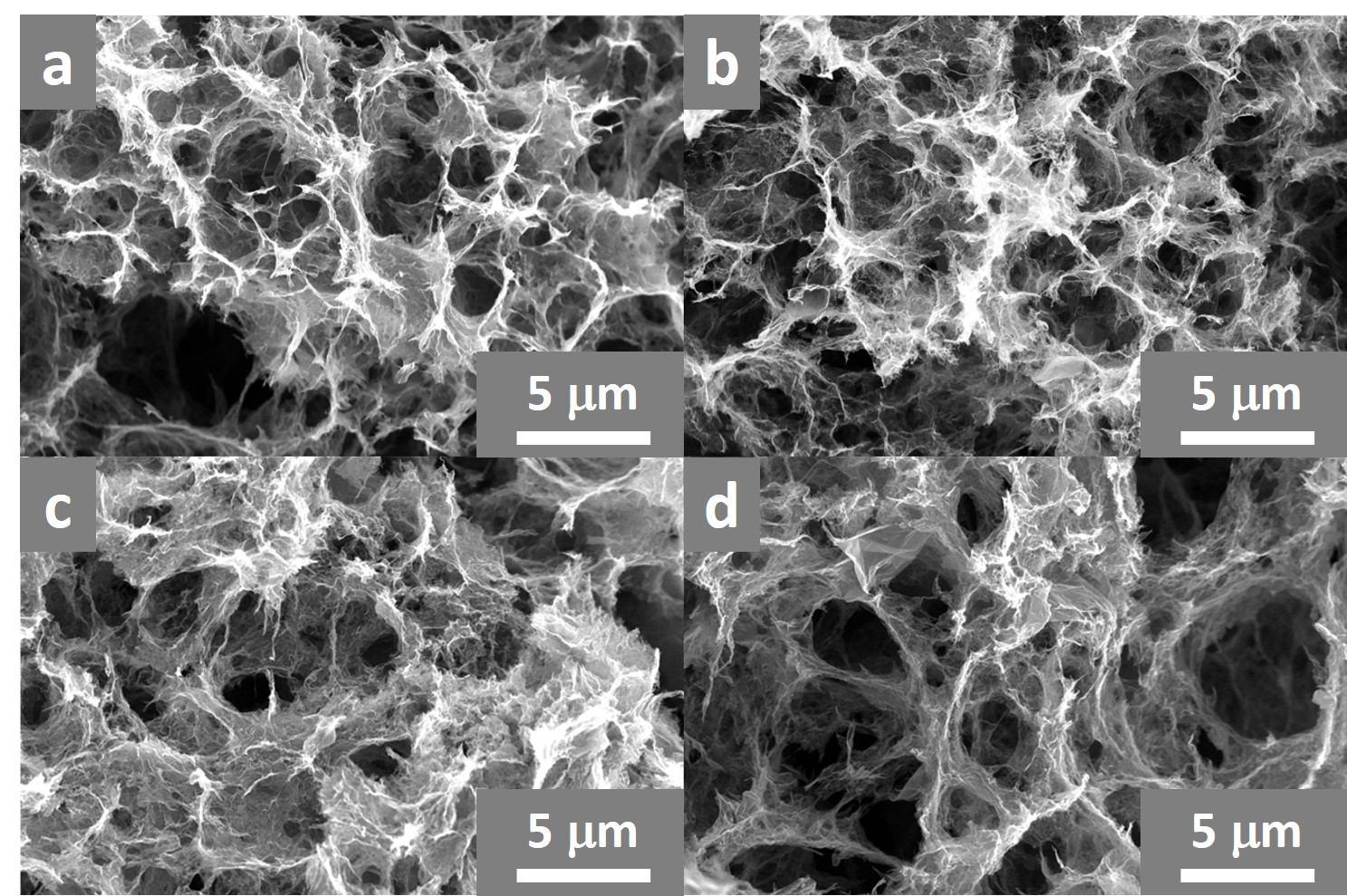


**Figure S22.** Low-resolution SEM images of NC aerogel (a), rNC_10s_ aerogel (b), rNC_30s_ aerogel (c), and rNC aerogel (d).


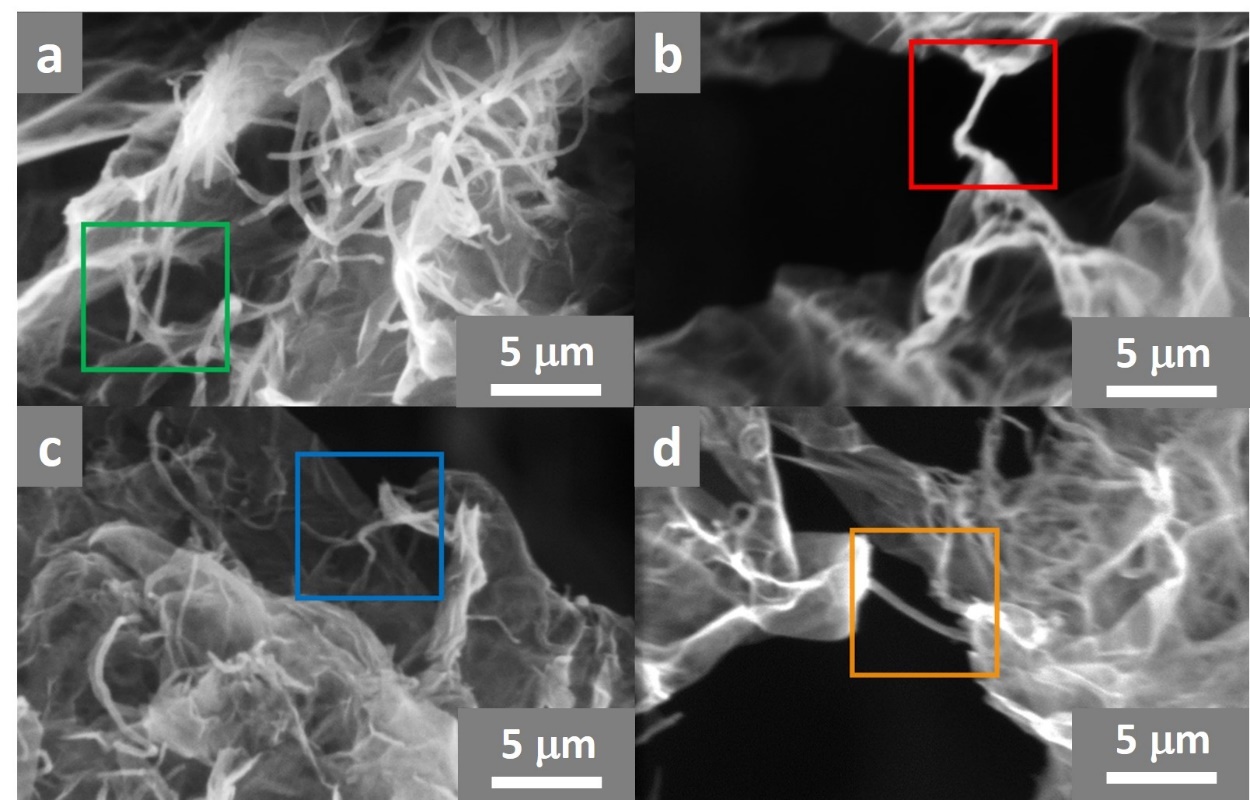


**Figure S23.** High-resolution SEM images of NC aerogel (a), rNC_10s_ aerogel (b), rNC_30s_ aerogel (c), and rNC aerogel (d).


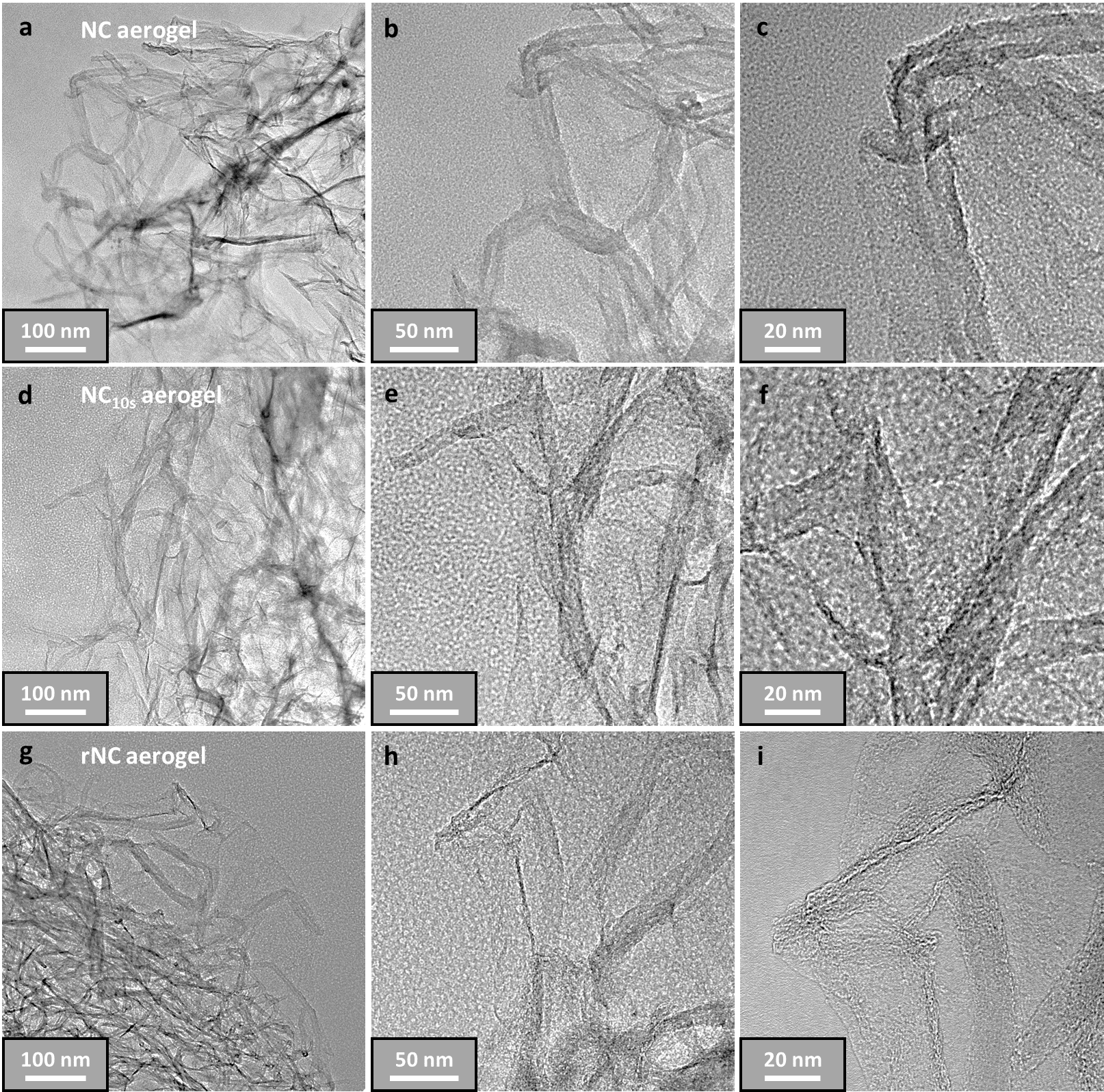


**Figure S24.** TEM images of NC aerogel (a-c), rNC_10s_ aerogel (d-f), and rNC aerogel (g-i).


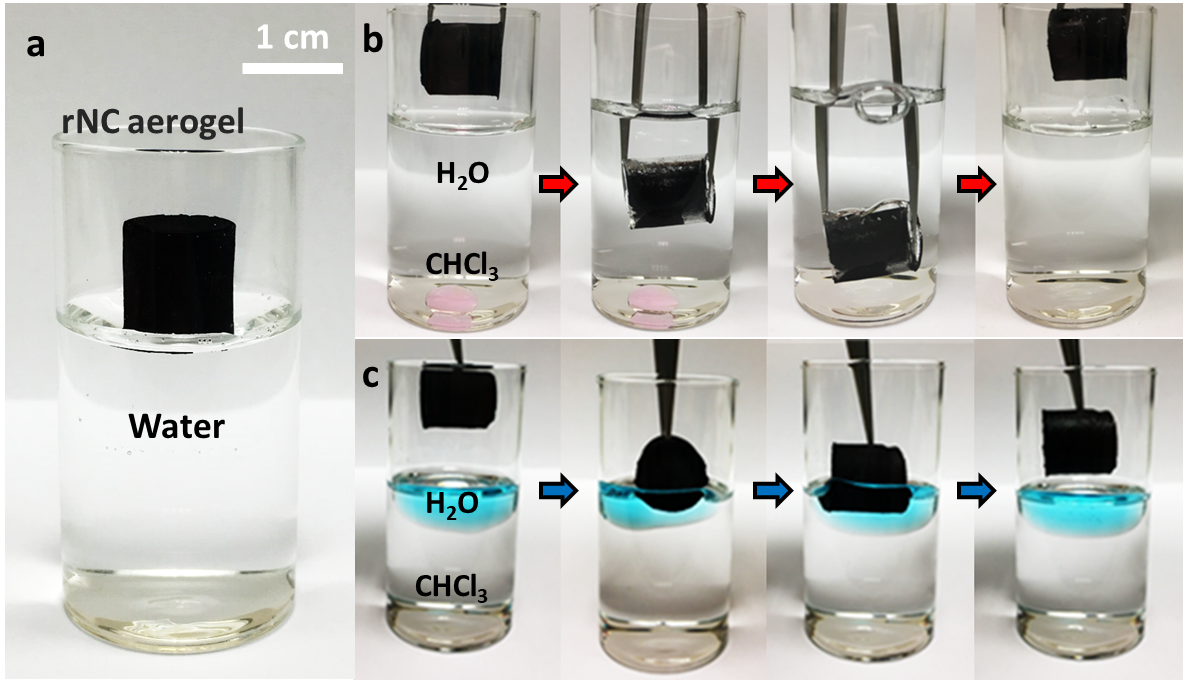


**Figure S25.** (a) Low-density rNC aerogel with superhydrophobic characteristics. (b) Digital photos showing prompt absorption of rhodamine B-contained CHCl_3_ phase on rNC aerogel from water. (c) Digital photos demonstrating no absorption of MB-contained water on rNC aerogel from the CHCl_3_ phase.


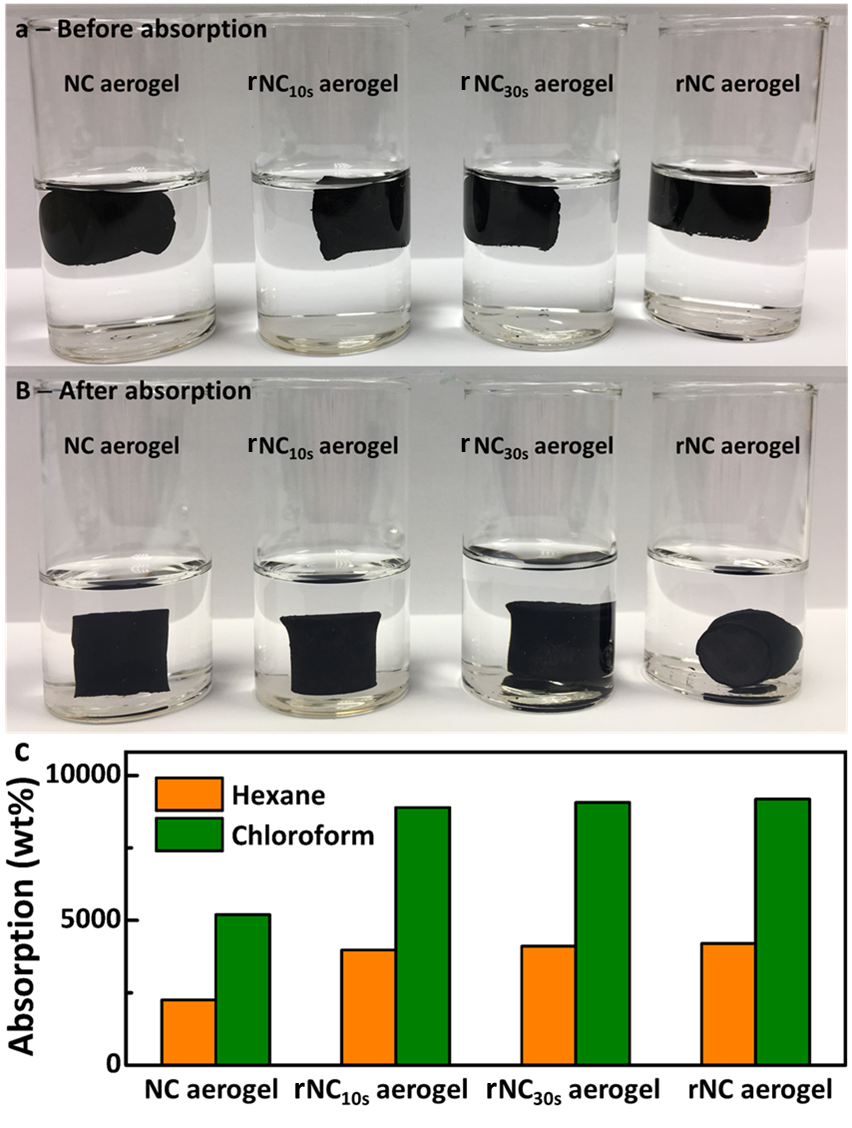


**Figure S26.** (a-b) Digital photos of chloroform absorption using NC aerogel, rNC_10s_ aerogel, rNC_30s_ aerogel, and rNC aerogel. (c) Hexane and chloroform absorption performance on as-discussed aerogels.

**
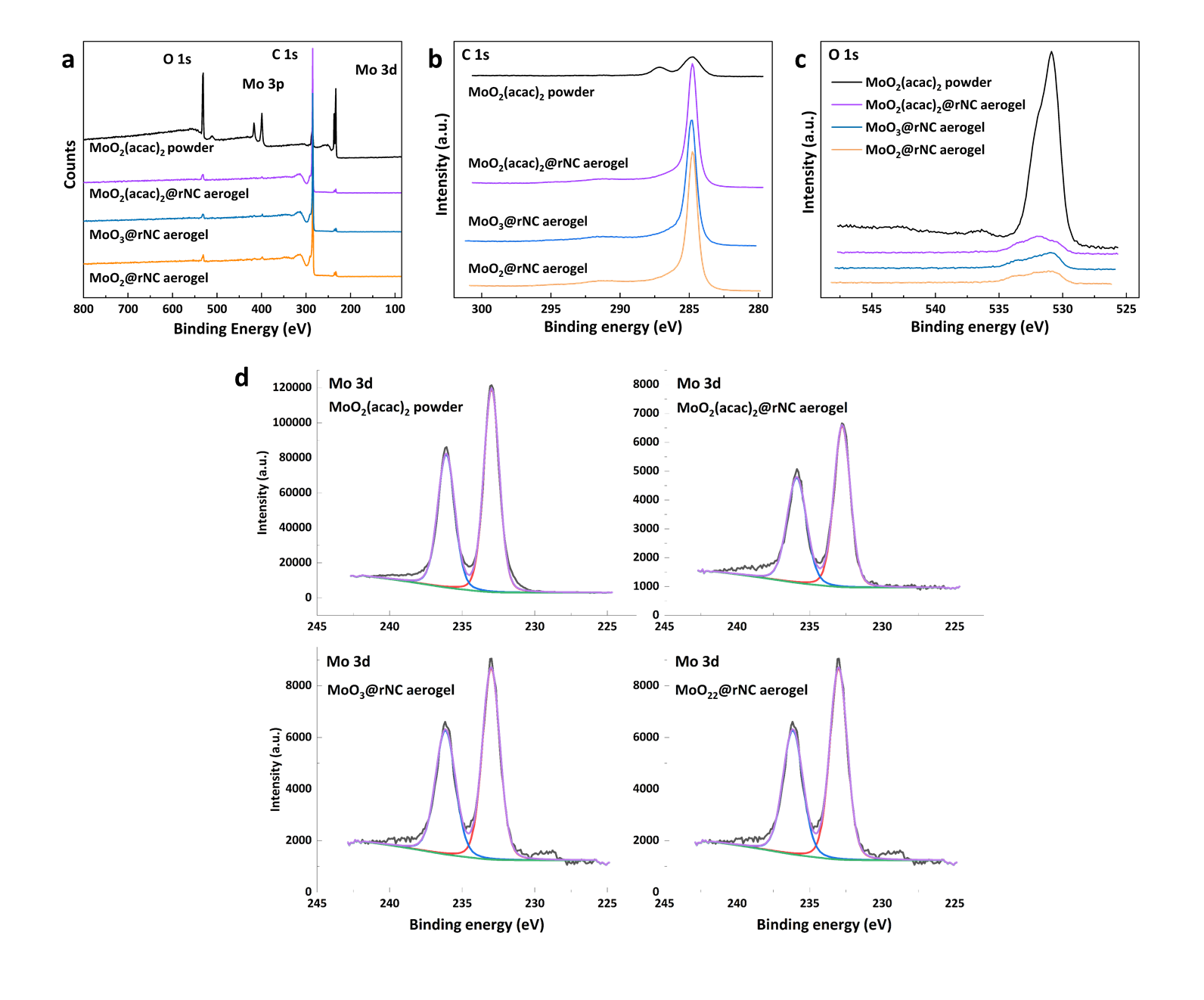
**

**Figure S27.** XPS full survey spectra (a), C1s region (b), O1s region (c) and Mo 3d region (d) of MoO_2_(acac)_2_ powder, MoO_2_(acac)_2_@rNC aerogel, MoO_3_@rNC aerogel, and MoO_2_@rNC aerogel.

**Table S4**. Microstructural parameters of NC aerogel and rNC aerogel.

| Aerogel name | SSA  (m^2^/g) | V_Meso-porosity_  (cm^3^/g) | V_Micro_  (cm^3^/g) | D_Mesopore_  (nm) |
| --- | --- | --- | --- | --- |
| NC aerogel | 477.1 | 2.16 | 0.0112 | 38.3 |
| rNC aerogel | 606.8 | 2.38 | 0.0187 | 41.3 |

Based on the prominent diffraction peak (110) from the XRD data, we selected the MoO_2_(110) surface as a model catalyst for nanoscale simulation. To gain insights into the influence of graphene on the electronic structure of the MoO_2_(110) surface (Figures S28a-S28c), DFT calculation was utilized to obtain the density of states of the pure MoO_2_(110) surface, graphene, and MoO_2_(110)@graphene. The MoO_2_(110) surface was derived from a fully relaxed MoO_2_ bulk. The calculated lattice parameters of bulk MoO_2_, which belongs to the P2_1_/c space group in the monoclinic crystal system, were determined as follows: *a* = 5.586 Å, *b* = 4.900 Å, and *c* = 5.671 Å, with a monoclinic angle *β* of 120.17°. In our experimental measurements, the lattice parameters were found to be *a* = 5.611 Å, *b* = 4.856 Å, and *c* = 5.629 Å, with a monoclinic angle *β* = 120.9°. The calculated lattice parameters are consistent with the experimental values, with an error of less than 1%.^[11]^ We successfully obtained the optimized structure of MoO_2_(110)@graphene (Figures S28d-S28f) with appropriate lattice constants (*a* = 7.46 Å, *b* = 11.28 Å) and an interlayer distance between graphene and the MoO_2_(110) surface (*d* = 4.21 Å). These results indicate the physical adsorption of the MoO_2_(110) surface on the graphene nanosheet via *van der* Waals forces.


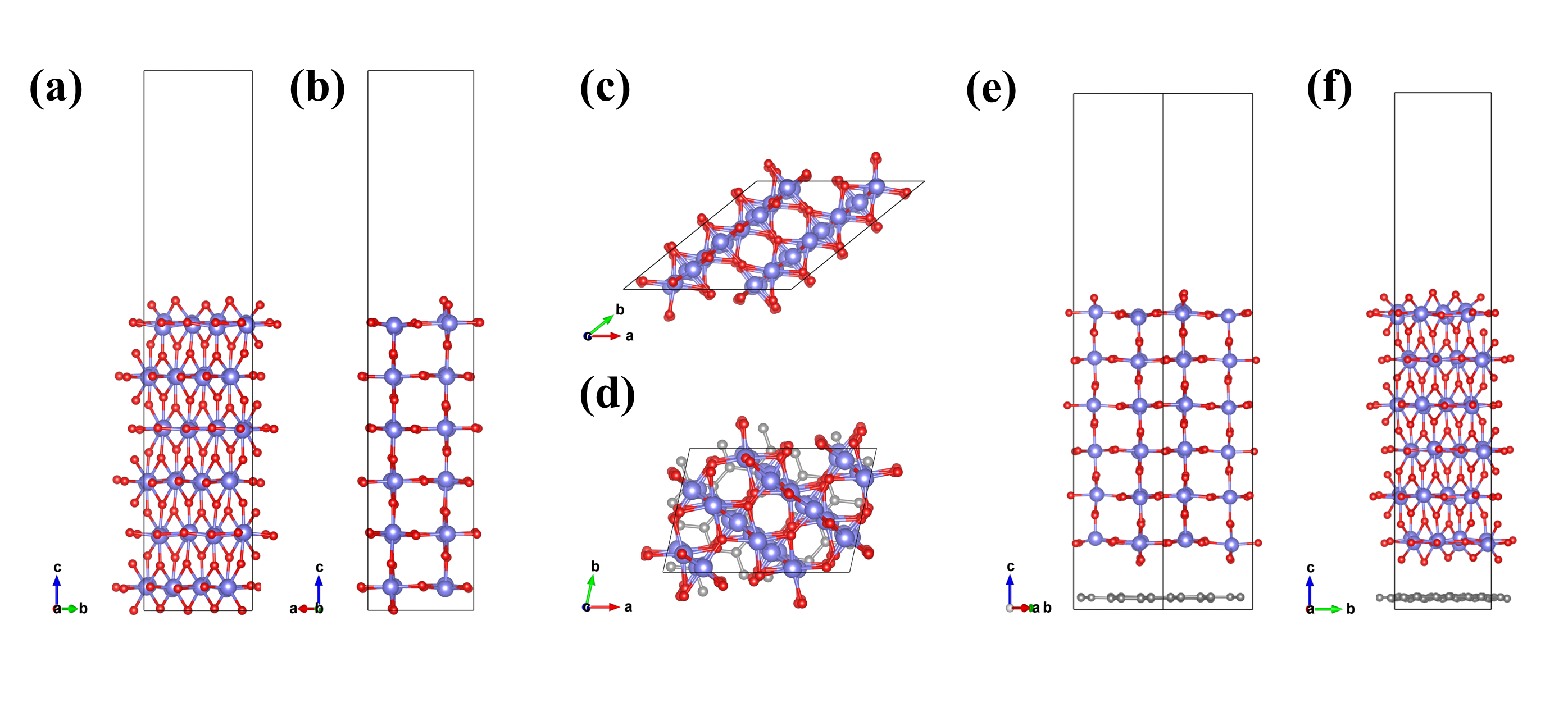


**Figure S28.** The crystal structures of MoO_2_ (110) surface (a), (b), and (c) along the a, b, and c axis. The crystal structures of MoO_2_@graphene (d), (e), and (f) along the c, a, and b axis.

The total density of states (TDOS) and partial density of states (PDOS) were calculated to analyze the interaction between the pure MoO_2_(110) surface, graphene, and MoO_2_(110)@graphene. Figures 6b-c present the TDOS comparison. All three systems, pure MoO_2_(110) surface, graphene, and MoO_2_(110)@graphene, exhibit excellent metallicity in their TDOS. The PDOS analysis illustrated that the main DOS contributions for Mo and O in the MoO_2_(110) surface originate from their respective 4*d* orbitals and 2*p* orbitals. Conversely, the predominant DOS for C in graphene was attributed to its 2*p* orbitals. Comparing the PDOS of Mo and O in the MoO_2_(110) surface with those in the MoO_2_(110)@graphene interface, a downward shift along the Fermi level was observed. This shift indicates electron transfer from graphene to Mo and O in the MoO_2_(110)@graphene system. This result was consistent with the increased electron density observed on Mo atoms in Figure 6e (see manuscript). The yellow regions around O at the bottom layer of MoO_2_(110)@graphene indicate an increase in electron density, while the corresponding blue regions on C from graphene (delocalized π bonds) indicate electron depletion. In addition, the surplus electrons can migrate to adjacent Mo atoms, which can also be observed in the interior atoms of the thick MoO_2_ slab. These excess electrons may arise from the overlap of inner crystal orbitals, resulting in electron injection. Although the bottom layer of Mo atoms contains loosely bound electrons, the value of the isosurface is lower than the observed electron count.

To further reveal the charge transfer between graphene and MoO_2_(110), the work function (W_F_) for both materials is determined using Equation S5:

$W_{F}= E_{vac}- E_{F}$ Equation S5

Here, *E*_vac_ represents the energy of the electron in vacuum, and *E*_F_ expresses the energy value of the Fermi level. *W*_F_ values for graphene and MoO_2_(110) are 5.51 eV and 4.25 eV, respectively.

The difference in work function between graphene and MoO_2_(110) verifies that electrons will spontaneously transfer from electron-rich graphene to MoO_2_(110), thereby enhancing the total conductivity of the graphene-MoO_2_(110) interface. This electron transfer strengthens the ability of MoO_2_(110) to donate electrons during the catalytic process. Additionally, considering the interaction between graphene and MoO_2_(110), it is observed that the Mo-O bond length at the bottom layer in MoO_2_(110) shortens from a range of 1.95~2.14 Å to 1.77~2.11 Å. This significant change suggests that the excellent catalytic capacity exhibited by the MoO_2_@rNC catalyst can be accredited to a higher degree of electron transfer from graphene to the MoO_2_ nano-catalysts.


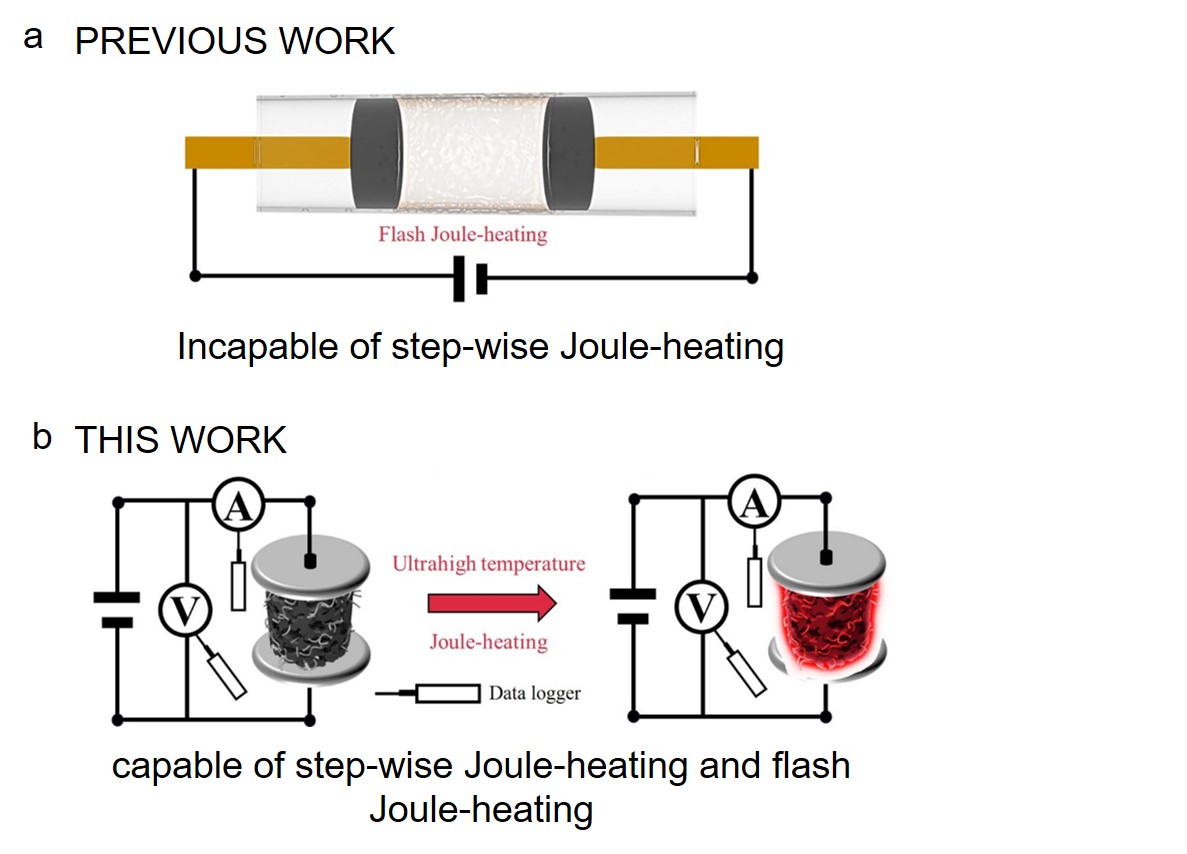


**Figure S29.** Figure R1. Schematic illustrations of (a) the Joule-heating setup for the flash-synthesis of graphene (adapted from Luong *et al*.) and (b) the ultrahigh temperature Joule-heating setup designed for 3D bulk nanocarbon aerogels (adapted from Xia *et al*.).

Despite the flash Joule-heating methodology also has attracted ever-growing attention, while which adopts a completely different Joule-heating setup to induce the Joule-heating effects, compared to the ultrahigh temperature Joule-heating setup designed for NC aerogels in this work. The Joule-heating setup difference between the flash Joule-heating and the ultrahigh temperature Joule-heating setup is illustrated in Figure S29.


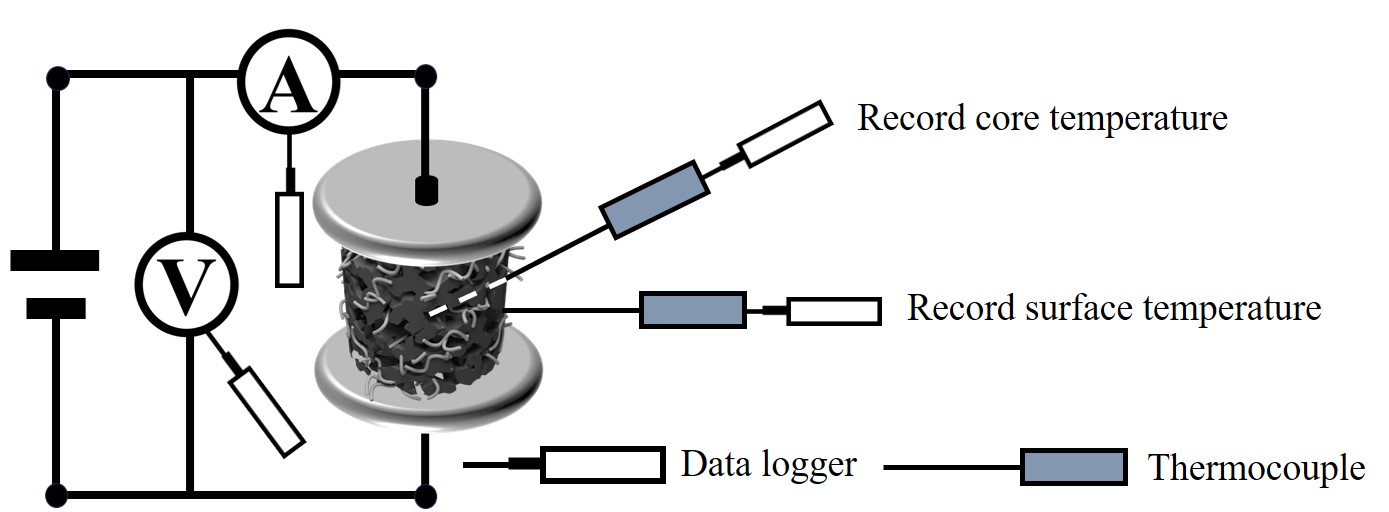


**Figure R30.** Schematic demonstration of conducting Joule-heating experiments using the proposed Joule-heating system.


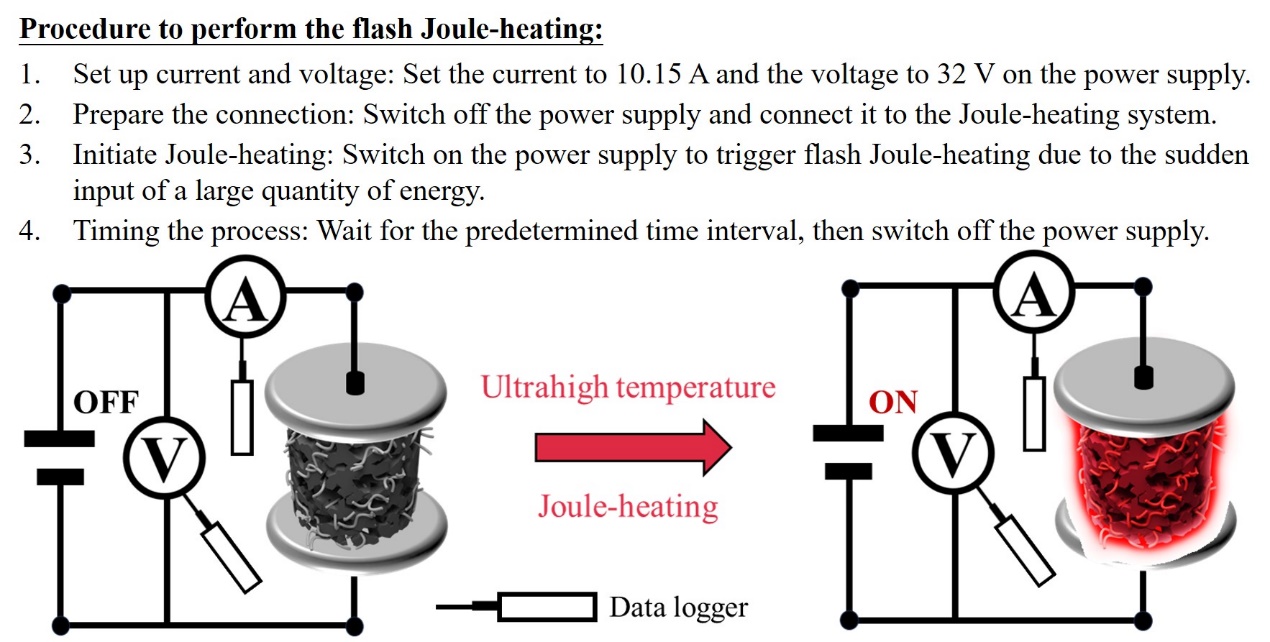


**Figure 31.** Schematic illustration of conducting flash Joule-heating experiments.

Procedure for performing flash Joule-heating:

1. Set up current and voltage: Set the current to 10.15 A and the voltage to 32 V on the power supply.
2. Prepare the connection: Switch off the power supply and connect it to the Joule-heating system.
3. Initiate Joule-heating: Switch on the power supply to trigger flash Joule-heating due to the sudden input of a large quantity of energy.
4. Timing the process: Wait for the predetermined time interval, then switch off the power supply.

**References:**

37. Kresse, G.; Furthmüller, J., *Comput. Mater. Sci.* **1996,** *6* (1), 15-50.

38. Kresse, G.; Furthmüller, J., *Phys. Rev. B* **1996,** *54* (16), 11169-11186.

39. Perdew, J. P.; Burke, K.; Ernzerhof, M., *Phys. Rev. Lett.* **1996,** *77* (18), 3865-3868.

40. Grimme, S.; Ehrlich, S.; Goerigk, L., *J. Computat. Chem.* **2011,** *32* (7), 1456-1465.

41. Blöchl, P. E., *Phys. Rev. B* **1994,** *50* (24), 17953-17979.

42. Ji, J.; Aleisa, R. M.; Duan, H.; Zhang, J.; Yin, Y.; Xing, M., *iScience* **2020,** *23* (2), 100861.

43. Momma, K.; Izumi, F., *J. Appl. Crystallogr.* **2011,** *44* (6), 1272-1276.

44. ISO 14040 International Standard 14040. Environmental Management - Life Cycle Assessment - Principles and Framework, International Organisation for Standardization, Geneva, Switzerland (2006)

45. ISO 14044 International Standard 14044 Environmental Management - Life Cycle Assessment - Requirements and Guidelines, International Organisation for Standardization, Geneva, Switzerland (2006)
